# Mechanotransduction: Exploring New Therapeutic Avenues in Central Nervous System Pathology

**DOI:** 10.3389/fnins.2022.861613

**Published:** 2022-04-28

**Authors:** Daniela Nogueira Rocha, Eva Daniela Carvalho, João Bettencourt Relvas, Maria José Oliveira, Ana Paula Pêgo

**Affiliations:** ^1^Instituto de Engenharia Biomédica (INEB), Universidade do Porto, Porto, Portugal; ^2^Instituto de Investigação e Inovação em Saúde (i3S), Universidade do Porto, Porto, Portugal; ^3^Faculdade de Engenharia (FEUP), Universidade do Porto, Porto, Portugal; ^4^Instituto de Biologia Molecular e Celular (IBMC), Universidade do Porto, Porto, Portugal; ^5^Departamento de Biomedicina, Faculdade de Medicina, Universidade do Porto, Porto, Portugal; ^6^Instituto de Ciências Biomédicas Abel Salazar (ICBAS), Universidade do Porto, Porto, Portugal

**Keywords:** mechanobiology, extracellular matrix, mechanotransduction, central nervous system, neurodegenerative disorders

## Abstract

Cells are continuously exposed to physical forces and the central nervous system (CNS) is no exception. Cells dynamically adapt their behavior and remodel the surrounding environment in response to forces. The importance of mechanotransduction in the CNS is illustrated by exploring its role in CNS pathology development and progression. The crosstalk between the biochemical and biophysical components of the extracellular matrix (ECM) are here described, considering the recent explosion of literature demonstrating the powerful influence of biophysical stimuli like density, rigidity and geometry of the ECM on cell behavior. This review aims at integrating mechanical properties into our understanding of the molecular basis of CNS disease. The mechanisms that mediate mechanotransduction events, like integrin, Rho/ROCK and matrix metalloproteinases signaling pathways are revised. Analysis of CNS pathologies in this context has revealed that a wide range of neurological diseases share as hallmarks alterations of the tissue mechanical properties. Therefore, it is our belief that the understanding of CNS mechanotransduction pathways may lead to the development of improved medical devices and diagnostic methods as well as new therapeutic targets and strategies for CNS repair.

## Introduction

Mechanotransduction is the process by which a cell translates mechanical stimuli into biochemical signals. The transduced signals can vary in their properties: electrical, involved in the depolarization of cellular membranes; chemical, triggering of second messengers; or transcriptional, as in the activation of gene expression; among others. Mechanotransduction is ubiquitously present in several taxonomic groups such as *Eubacteria, Archaea* and *Eukarya*, suggesting an early evolutionary occurrence of mechanotransducers, which advocates an important role for this process in living organisms.

The concept and importance of mechanotransduction have been initially identified on cells that typically experience mechanical stimuli *in vivo*, like mesenchymal and epithelial cells, as well as on sensory cells, like the inner ear hair cells (Gospodarowicz et al., 1978; Bissell et al., 1982; Vlodavsky et al., 1982; Davies et al., 1992, 1997; Wang et al., 1993). Today it is known that mechanical forces influence the growth, shape and behavior of nearly every cell, tissue and organ of the human body. Cells can sense and respond to a wide range of external chemical and physical signals and, consequently, change their morphology, dynamics and behavior. As such, mechanotransduction has become a topic of increasing awareness and consequently scientific interest in many fields of research. A vast body of research has shown that mechanical forces are ubiquitous *in vivo* and can directly impact cell function.

Initial studies of mechanotransduction in the nervous system were performed with sensory cells—the somatosensory neurons. In mammals, detection of mechanical forces by the somatosensory system is performed by primary afferent neurons, which can detect a wide range of mechanical stimuli (Delmas et al., 2011). Psycho-physical techniques have established in 1882 that sensory spots, defined as regions of low threshold to a given kind of stimuli, exist in the human skin. Cutaneous somatosensory receptors detect a wide range of mechanical stimuli, including light brush of the skin, texture, vibration, touch and noxious pressure (Delmas et al., 2011).

One of the main challenges in the study of sensory systems is to discover the basis of the transduction process. Rhodopsin, the light-transducing molecule, has been known for 130 years, and the olfactory receptors were discovered 20 years ago (Buck and Axel, 1991). However, molecules that transduce physical forces as osmotic force, touch, vibration, and texture, have been more difficult to identify. Corey and Hudspeth (Corey and Hudspeth, 1979) have shown that neurosensory transduction is extremely rapid. Using hair cells they observed that the movement of a hair bundle produced an electrical response within 40 μs. Neurosensory mechanotransduction has been revised elsewhere [see reference Delmas et al. (2011) for additional information] and will not be, therefore, further explored.

This review will focus on the role of mechanical sensing in the central nervous system (CNS) in the context of disease, particularly highlighting the contribution of the mechanical properties of the extracellular matrix (ECM) to this process. Although there are some hints on how forces impact these regulatory functions, clarifying these mechanisms remains crucial for a better understanding of neuromechanics. This could lead to alternative prognostic and therapeutic options that can, in the future, improve tissue repair and regeneration.

## Mechanotransduction in Central Nervous System Pathology

Tissue architecture reflects a balance in which cells adapt their cytoskeletal tension to match the forces generated by neighboring cells and the surrounding ECM, and the disruption of this equilibrium can contribute to a variety of diseases. Many times, it is unclear whether mechanical changes at tissue level are an early cause of disease, a mechanism of progression or a late symptom. One of the first diseases to be associated with biomechanics and mechanotransduction was atherosclerosis, for which it has been shown that low-oscillatory shear stress correlates with sites of atherosclerotic plaques (Lehoux and Tedgui, 2003). Since then, many other diseases have been correlated with mechanotransduction alterations (Table 1).

**TABLE 1:** Analysis of several CNS pathologies and their impact in tissue structure.

Disorder	Tissue stiffness	ECM remodeling	Signaling pathway involved	Possible therapeutic target	References
Retinal Detachment	↓	Yes	Hippo EGFR Notch	RPE pumps	Sivak and Fini, 2002; Morante and Desplan, 2004; Dupont et al., 2011; Chou and Siegel, 2012;
Glaucoma	↑	Yes	Hippo	Optic nerve head mechanics	Rosario Hernandez and Pena, 1997; Heijl et al., 2002; Kass et al., 2002; Dupont et al., 2011
Migraine	=	Yes (mild)	PKC MMP activity	CNS-blood vessels crosstalk	Goadsby et al., 1990; Di Castro et al., 2006; Martins-Oliveira et al., 2009; Nassini et al., 2010; Lakhan and Avramut, 2012
CNS injury	↓	Yes	PKC Rho/ROCK MAP kinase	BBB permeability	Krizbai and Deli, 2003
	↓	Yes	EGFR Rho/ROCK	Glia architecture	Morante and Desplan, 2004
	↓	Yes	ERK PKC	MMP activity	Hsieh et al., 2010
	↓	Yes	Rho/ROCK PKC	CSPGs	Morgenstern et al., 2002
Alzheimer’s	↓	Yes	Rho/ROCK PLA_2_	Aβ fibrils	Moses et al., 2006
	↓	Yes	Rho-ROCK	ROCK	Jagielska et al., 2012; Hensel et al., 2015
	↓	Yes	ERK PKC	MMP activity	Hsieh et al., 2010
Parkinson	↓	Yes	Rho/ROCK pathway	ROCK	Jagielska et al., 2012; Hensel et al., 2015
	↓	Yes	ERK PKC	MMP activity	Hsieh et al., 2010
Multiple Sclerosis	↓	Yes	Rho-ROCK	ROCK	Jagielska et al., 2012; Hensel et al., 2015
	↓	Yes	ERK PKC	MMP activity	Hsieh et al., 2010
Cancer	↑	Yes	FAK signaling Rho/ROCK signaling	Talin-1 MMP activity	Ulrich et al., 2009; Sen et al., 2012

*↑, increased stiffness; ↓, decreased stiffness; =, unchanged stiffness; EGFR, Epidermal growth factor receptor; RPE, retinal pigment epithelium; PKC, protein kinase C; CNS, central nervous system; ROCK, Rho-associated protein kinase; BBB, brain blood barrier; ERK, extracellular signal–regulated kinase; MMP, matrix metalloproteinase; CSPG, chondroitin sulfate proteoglycan; PLA_2_, Phospholipase A2; FAK, Focal adhesion kinase.*

Central nervous system tissues are among the softest tissues in the body and, due to their mechanical fragility, are particularly susceptible to mechanical damage caused by trauma. As such, brain and spinal cord are protected by stronger structures such as the pia mater, the dura mater, the skull, and the vertebras. In some neurodegenerative diseases a change in stiffness of the affected tissues is partly accountable for cell physiological functions loss (Moshayedi et al., 2010). Studies on the mechanical properties of glia have demonstrated an alteration of CNS tissue stiffness either as a result of CNS disorders (Freimann et al., 2011; Murphy et al., 2012, 2016; Schregel et al., 2012; Hain et al., 2016; ElSheikh et al., 2017) or of traumatic injuries (Saxena et al., 2012). Distinct CNS disorders, described to be intimately related with mechanotransduction properties alterations, will be addressed in the following sections.

### Headache

Pain can be evoked by mechanical stimuli and inflammatory conditions (Di Castro et al., 2006). As such, increased mechanosensitivity has been considered to play a role in the pathophysiology of headache and of neuropathic pain (Di Castro et al., 2006; Nassini et al., 2010). Migraine headaches, which are the most common type of primary headaches, are described as neurovascular disorders affecting up to 15–20% of the world population (Edvinsson and Uddman, 2005). Migraine is characterized by attacks of moderate to severe headache that last from 4 to 72 h, often unilateral, pulsating and associated with photophobia/phonophobia and/or nausea/vomiting (Edvinsson and Uddman, 2005) with one-third of the patients having associated symptoms of neurological aura (Ferrari, 1998).

Several studies have found elevated hypertension and dyslipidemia in migraineurs (Gardener et al., 2016; Finocchi and Sassos, 2017), therefore suggesting a role of vasodilation of cerebral and/or meningeal blood vessels on migraine, which correlates with mechanosensitivity. Calcitonin-related proteins, known to be coupled to protein kinase C (PKC) pathway, have been shown to play a role in this mechanical hypersensitivity in migraine (Di Castro et al., 2006; Nassini et al., 2010).

There has been an extensive debate on whether headaches have a vascular or neurogenic origin (Bahra et al., 2001). Nonetheless, headache is also a common symptom after lumbar puncture, which has been associated with intracranial hypotension due to cerebrospinal fluid (CSF) leakage (Lahoria et al., 2012). Therefore, the genesis of this disorder is believed to be in the CNS and not a vascular cause, as vascular changes appear to be a consequence of the neural mechanisms and not the initiator (May et al., 2000). In fact, some populations of neurons suspected of transducing neuronal signals into vasomotor responses have been identified in the cerebral cortex (Edvinsson and Uddman, 2005; Mikhailov et al., 2017). These findings suggest that neurons emit projections toward blood vessels in specific brain regions, and that blood vessels have the ability to respond to neurotransmitters, by modifying their diameter and consequently local blood flow.

These results hint a central role to the interaction between the CNS and the surrounding blood vessels, with mechanotransduction being a mechanism of disorder progression and, as such, a potential therapeutic target. In line with this, it was recently proved that the mechanisms underlying the beneficial effects of migraine treatment with botulinum toxins were related with mechanotransduction pathways (Paterson et al., 2014).

The signaling pathways involved in this process are further explored in section “The Extracellular Matrix Role in the Process of Mechanotransduction.”

### Eye Disorders

**FIGURE 1:**
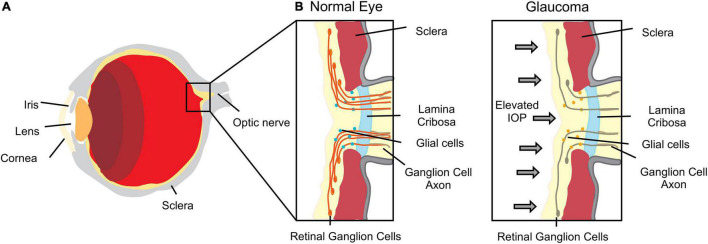
Scheme of an eye. **(A)** Major anatomical structure and organization. **(B)** Enlarged section of the lamina cribrosa and optic nerve representation of tissue deformation in response to increased intraocular pressure (IOP) representative of glaucomatous optic neuropathy.

Changes in topography and/or stiffness of eye structures have also been correlated with pathological conditions (Morgan et al., 2013). The optic nerve head constitutes an interesting biomechanical structure with complex load-bearing tissue architecture, the *lamina cribrosa*, which is subjected to intraocular pressure stress (Morgan et al., 2013; Strouthidis and Girard, 2013; Figure 1). The biomechanical properties of the sclera are also extremely important as it plays a pivotal role in controlling eye shape during events that promote eye deformation such as movement, accommodation and remodeling. In fact, the ECM and cellular constituents of the sclera contribute to the biomechanical environment that enables the sclera to accomplish these requirements.

Strouthidis and Girard (2013) suggested that optic nerve biomechanics is the “link” by which intraocular pressure (IOP) can affect other factors such as ischemia, inflammation, autoimmunity and glial cell biology. This biomechanical theory explains why some people are predisposed to develop glaucomatous optic neuropathy, independently of their IOP levels. Moreover, the increasing predisposition related to aging may also be explained by changes in biomechanical behavior through alterations of the connective tissue (Strouthidis and Girard, 2013). In glaucomatous optic neuropathy, one of the leading causes of blindness worldwide, IOP causes eye tissue stress, deformations and strain, leading eventually to damage and loss of retinal ganglion cell axons (Sigal and Ethier, 2009; Figure 1). IOP reduction remains the only therapy used to preserve vision and retard glaucomatous progression (Huang et al., 2017). This suggests that the biomechanical effects of IOP to the tissues surrounding the optic nerve head are central to disease (Sigal, 2009). Therefore, the correct tuning of optic nerve head mechanics may constitute a potential therapeutic target in glaucomatous optic neuropathy.

Biomechanical properties may also affect ocular growth. Changes in sclera mechanical properties have been documented during development and pathological conditions as the cause of abnormal ocular growth such as myopia, where sclera becomes thinner, weaker and more extensible (McBrien et al., 2009). Mechanical properties of the eye structures have also been shown to play an important role on retinal detachment. Chou and Siegel (2012) have developed a mathematical model to investigate the relationship between flows and pressures and retinal detachment. This study has shown that the destabilization of retinal pigment epithelium (RPE) *per se* is not enough to induce large-scale delamination. Instead, spontaneous uniform tissue separation occurs when RPE pumps fail and the adhesion energy retina-RPE is globally reduced. Furthermore, it has also been suggested that retina elastic modulus is decreased in conditions such as retinal detachment (Davis et al., 2012). Observation corroborated by thermodynamic calculations has demonstrated that supported membranes, like the normal retina, have greater stiffness values than unsupported membranes (Agrawal and Radhakrishnan, 2009). Another study from Davis et al. (2012) has found a significant influence in gene regulation of Müller cells as a function of substrate stiffness. This study emphasizes the role of YAP (Yes-associated protein) and TAZ (transcriptional coactivator with PDZ-binding motif) as important therapeutic targets for retina and optic nerve pathologies. These had previously been identified as nuclear relays of mechanical signals exerted by ECM rigidity and cell shape (Dupont et al., 2011). Overall, these studies suggest that eye mechanical properties and its mechanotransducers and mechanosensors, are key therapeutic targets in distinct eye pathologies. Nevertheless, although some clinical procedures already exploit mechanical forces like vitrectomies, laser photocoagulation or pneumatic retinopexy, deeper knowledge of mechanotransduction mechanisms is required for further therapeutic developments.

### Neurodegenerative Disorders

The incidence of neurodegenerative disorders is increasing worldwide, particularly within the aged population. So far, our understanding of the nature and origins of these disorders is still limited, as most neurodegenerative disorders are heterogeneous. Nevertheless, intense efforts are being performed to achieve a better understanding of these conditions.

Alzheimer’s disease is a chronic disorder characterized by cerebrovascular inflammation, accumulation of senile amyloid plaques in the brain, formation of neurofibrillary tangles and, ultimately, neuronal loss. Alzheimer’s disease is one of the most studied neurodegenerative diseases in terms of mechanobiology. The deposition of amyloid plaques, structures formed by stable fibrillar aggregates and unstable and transient assemblies of amyloid beta (soluble oligomers), constitutes a substantial alteration to the brain mechanical properties. Consequently, glial cells sense these stiff plaques and adjust their mechanotransduction responses (Hall et al., 2021).

The deposition of amyloid fibrils is associated with several other neurodegenerative diseases, as Parkinson’s disease and neurodegenerative processes associated to type-2 diabetes. Amyloid fibrils are highly ordered nanoscale assemblies of protein protofibrils composed predominantly of β-sheet structures. These have been found to alter cell membrane properties, such as fluidity, and molecular architecture, leading to neurovascular dysfunction and chronic degeneration (Yang et al., 2010). Interestingly, amyloid fibrils (Aβ fibers) deposits are correlated with the activation of phospholipase A2 (PLA_2_) (Moses et al., 2006), which is known as a crucial modulator of membrane properties in health and disease. In Alzheimer’s, brain reduced membrane fluidity has been directly associated with decreased PLA_2_ activity (Ross et al., 1998; Eckert et al., 2000). Amyloid fibrils were shown to have stiffness values comparable to cytoskeleton components such as actin filaments, microtubules or intermediate filaments (Sweers et al., 2012). Several authors have been exploring amyloid fibrils mechanical properties, behavior and stability, as the understanding of these features may shed light on the fundamental mechanisms of formation and structure dynamics of these nanostructures. Depending on the peptide length of its monomers, Aβ fibers will present different rupture forces. In fact, Xu et al. (2010) have stated that longer amyloid fibrils are more stable mainly due to their close contact and denser structure, suggesting that size may imply pathological consequences. Additionally, Paparcone et al. (2010) demonstrated that salt bridges contribute to stability, geometry and mechanical behavior of amyloid fibrils. Side chain interactions are described to influence the aggregation rate, as well as the chemistry and the mechanics of these fibrils.

Nonetheless, in comparison with unstable amyloid beta oligomers, amyloid fibrils are less cytotoxic. The oligomers structures create membrane pores in the targeted cells and are responsible for dysregulating calcium signaling, oxidative stress and alterations on the biophysical properties of cell membranes by rapidly decreasing their stiffness (Ungureanu et al., 2016).

Apart from amyloid plaques, the formation of neurofibrillary tangles is another hallmark of Alzheimer’s disease and even constitutes a better predictor of cognitive dysfunction in patients. These structures are formed by the hyperphosphorylation of the tau protein in brain cells, strongly disrupting the microtubules of neurons, modifying the axon mechanical properties (DeTure and Dickson, 2019).

**FIGURE 2:**
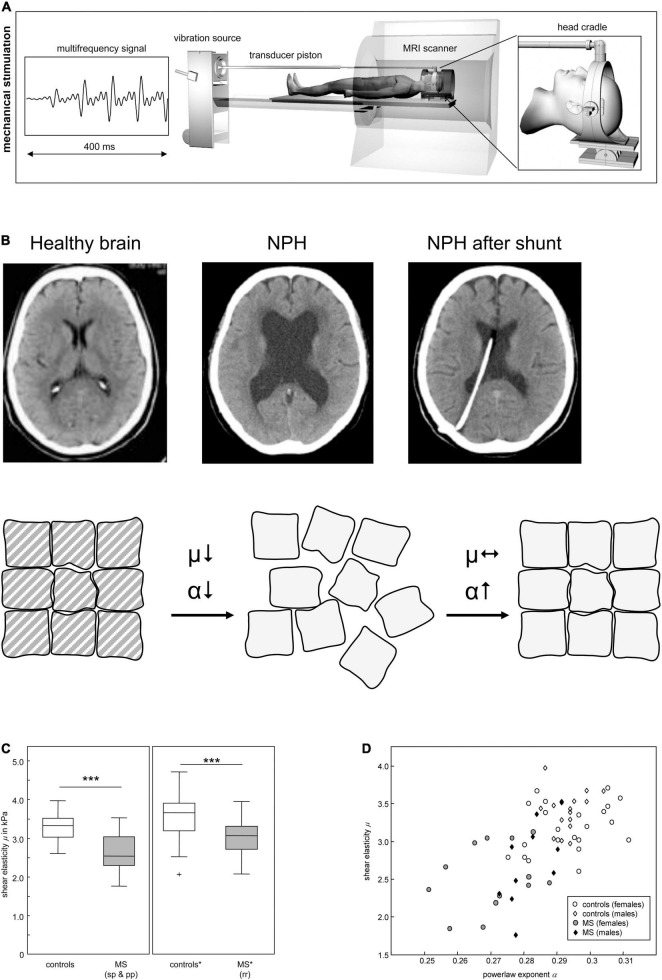
Mechanical properties in neurodegeneration. **(A)** Scheme of cerebral multifrequency MRE. The MRI scanner is combined with a device for acoustical head stimulations comprising a signal generator that produces a multifrequency signal composed from four harmonic frequencies of 25, 37.5, 50 and 62.5 Hz; a loudspeaker to generate acoustic vibrations; an extended piston that transfer the vibrations into the scanner and a head cradle to stimulate head vibrations mainly along the head-feet direction. **(B)** Cerebral MRE of NPH brains reveals a disease related decreased stiffness (μ), which is not recovered after surgical treatment. In contrast α increases after 3 months, to almost symptomatic values, suggesting that the topology of the tissue’s matrix is reorganized although its strength remains diminished. Reprinted with kind permission from Springer Science and Business Media from Freimann et al. (2011). **(C)** Reduction of brain elastic properties in healthy volunteers and multiple sclerosis (MS) patients. sp, secondary progressive; pp, primary progressive; rr, relapsing remitting. **(D)** Viscoelastic constants for prediction of brain pathology. Individual data of shear elasticity and power law exponent of brain of healthy volunteers and MS patients are represented. Panels **(A,C,D)** reprinted with kind permission from Springer Science and Business Media from Streitberger et al. (2012). Panel **(B)** reprinted with kind permission from Nature Portfolio from Yamada et al. (2019).

Hattori and coworkers (Hattori et al., 2011) have studied the diffusional properties of the corticospinal tract in patients with Alzheimer’s disease, Parkinson’s disease and idiopathic normal pressure hydrocephalus (iNPH) by diffusion tensor imaging (DTI). DTI became a popular Magnetic Resonance Imaging (MRI) technique to characterize microstructural changes in neuropathologies, as it enables the characterization of white matter fasciculi in three dimensions (3D). Many CNS pathologies influence tissue composition, architecture and alterations in diffusion of water within these tissues (Alexander et al., 2007). iNPH is a rare case of a neurological disorder whose symptoms may be relieved by surgical intervention, which mainly consists of implanting a ventriculoperitoneal shunt to drain excess cerebrospinal fluid. The success of these interventions is related with the mechanical re-organization of the brain tissue (Freimann et al., 2011; Figures 2B,C). Patients with iNPH present high white matter damage such as myelin loss and ischemia. Additionally, fractional anisotropy values and axial eigenvalues were significantly increased in these patients, suggesting alteration in the microstructure of the corticospinal tract, presumably a consequence of the mechanical pressure resulting from ventricular enlargement (Hattori et al., 2011). These microstructural alterations have been further correlated with tissue mechanical properties, using magnetic resonance elastography (MRE) (Freimann et al., 2011). MRE is a non-invasive and reproducible method that allows the evaluation of the mechanical properties of tissues and has been applied to assess biomechanical alterations of the living brain (Figure 2A). In general, patients with iNPH present lower tissue stiffness values, when compared with healthy controls (Figure 2B). Patients with Alzheimer’s disease also showed significantly softer brain parenchyma than matched controls (Murphy et al., 2016; Gerischer et al., 2018; Munder et al., 2018). Several processes may impact tissue mechanical properties in Alzheimer’s disease but, the knowledge on amyloid fibrils being several orders of magnitude stiffer than neurons and glia (Paparcone et al., 2010; Xu et al., 2010; Sweers et al., 2012) would, at a first glance, suggest increased stiffness of the patient brain tissue. Indeed, the decreased tissue stiffness may be a consequence of microstructural events that have destroyed cytoarchitectural integrity such as degradation/alteration of the ECM as well as the alteration of cell membrane stiffness by oligomers as previously referred. Moreover, this loss of microstructural integrity is in agreement with the findings of diffusion anisotropy studies showing increased brain anisotropy in Alzheimer’s patients (Hattori et al., 2011).

MRE has also been used by several authors to study the biomechanical properties of multiple sclerosis (MS) patients’ brains. The viscoelasticity and brain parenchymal volume of MS brains were shown to be reduced, both in human subjects and in animal models of MS (Riek et al., 2012; Schregel et al., 2012), in comparison with healthy controls (Figure 2D). However, measurements using other techniques have revealed contradictory data. A study published by Urbanski et al. (2019) stated that mechanical properties change throughout disease since acute or chronic demyelination exhibited opposite mechanical rigidities. Altogether, this data evidences that the above-mentioned pathologic conditions lead to significant changes in the tissues’ viscoelastic properties. Hence, MRE presents great potential as an additional tool to improve diagnostic sensitivity, as it provides a non-invasive and quantifiable “palpation” method of the brain. Biomechanical properties of the brain parenchyma alone may not be enough but, in combination with other biomarkers and with the clinical expression of the disease can lead to a better diagnostic method. Nevertheless, the question remains on whether mechanical changes are an early cause of disease, a mechanism of progression or a late symptom.

### Traumatic Central Nervous System Injury

Traumatic CNS injury results in disruption of the neural structures, local blood-brain barrier (BBB) disassembly and massive infiltration of immune cells. After the initial mechanical trauma (primary damage), cell damage is triggered and, within hours, the injury site and the surrounding hemorrhagic areas begin to undergo necrosis (secondary damage), recruiting more inflammatory cells and fibroblasts, which actively participate in ECM remodeling.

Microglia, the immune cells of the CNS, rapidly become activated and this state can prevail for years. These cells play a crucial role in the acute stages of the disease by clearing cellular and molecular debris within the injury site, constituting an important step toward restoration of homeostasis and regeneration. Nonetheless these activated cells also release toxic factors (such as reactive oxygen species, nitrogen species, glutamate, inflammatory cytokines, etc.) which with time may further damage the local environment. Consequently, microglia cells are even thought to be main contributors for the subsequent development of neurodegenerative disorders after TBI (Donat et al., 2017).

Along with microglia activation, astrocytes also change their phenotype from quiescent to activated, a process called reactive astrogliosis which ultimately leads to the formation of a glial scar. This changed environment is enriched in astrocytes, microglia and fibroblasts, as well as in cell debris and *de novo* produced ECM deposits. Even though the glial scar may provide several benefits such as the restoration of the BBB, prevention of a devastating inflammatory response and delimiting cellular degeneration and death, it also contributes to the establishment of a physical and chemical barrier to axonal regeneration (Pires and Pego, 2015).

Astrocytes, the most abundant cell type in the CNS, are the largest population of cells found in glial scars (Moshayedi et al., 2010). Astrogliosis is commonly characterized by increased expression of glial fibrillary acidic protein (GFAP), hypertrophy, hyperplasia, and increased ECM component production/secretion by astrocytes (particularly chondroitin sulfate proteoglycans—CSPGs—and laminin) (Fawcett and Asher, 1999; Morgenstern et al., 2002). The astrocytic response to CNS injury and disease has been the subject of several studies. Astrocytes naturally form a 3D meshwork/structure that extends throughout the brain which, as suggested by Mathewson and Berry (1985), is the ideal morphology for sensing mechanical disturbances in the parenchyma. It is possible that the observed astrocytic accumulation in the scar is, at least in part, the result of their mechanosensitive response. The ability of astrocytes to respond to mechanical stress could provide a general mechanism by which a variety of insults can be felt and managed in the CNS (Ostrow and Sachs, 2005), and could be extremely important for the development of future therapeutic strategies.

As previously mentioned, mechanotransduction events within the CNS have only been more recently studied, maybe because CNS tissues are not naturally subjected to intense mechanical stress as other structures in our body. Nevertheless, the spinal cord and some cranial nerves consistently experience physical stress during routine movements (Shreiber et al., 2009). Shreiber et al. (2009) studied the role of glia in CNS tissue and concluded that despite its individual stiffness, which is lower than neurons, glia significantly contributes to overall tissue stiffness and strength, working as cellular cross-linkers. Other authors (Georges et al., 2006; Lu et al., 2006; Jiang et al., 2007) have also highlighted the importance of glia architecture on its potential protective role against excessive tensile stress and strains, in order to limit injury. *In vitro* data suggests the impact of substrate stiffness on astrocytes development and possibly on their reactivity, generally augmented on stiffer substrates (Jiang et al., 2007; Moshayedi et al., 2010; Rocha et al., 2015; Wilson et al., 2016). Moreover, after the correlation of CSPGs with inhibition of axonal outgrowth and oligodendrocyte precursor cell (OPC) maturation (Back et al., 2005; Sloane et al., 2010), it has been established that treatment with chondroitinase ABC (chABC) can revert the inhibitory effects of CSPGs toward neural cells both *in vitro* (Steinmetz et al., 2005; Rocha et al., 2015) and *in vivo* (Bradbury et al., 2002; Caggiano et al., 2005). This treatment, breaks down CSPGs produced in response to injury in an attempt to recover the initial ECM environment both chemically and mechanically, emphasizing the importance of ECM structure and mechanical properties. Additionally, microglia cells are also responsive to mechanical properties changes by altering their gene and protein expression (Keating and Cullen, 2021). Although traditionally only studied in disease context, research has shown that glial cells play a pivotal role in health and disease. Furthermore, these cells can possess a dual role, as the glial scar represents a physical barrier to regeneration but at the same time is crucial to protect the CNS from further damage when everything else fails. As such, it is important to focus more on glial cell health, as well as on its response and behavior in the CNS.

### Cancer

Cancer is a disease characterized by the dysregulation of the cell cycle, particularly of signaling pathways that control proliferation and apoptosis. CNS tumors are divided into several categories: medulloblastomas, astrocytomas, oligodendrogliomas and ependymomas. Since gliosarcoma is a rare glioblastoma variant with scarce information available, it will not be included in this review.

Meningiomas are the most common CNS tumors, constituting 25% of all primary intracranial cancers and being more frequent in middle-aged and elderly patients. Most of the meningiomas are slow-growing tumors, which are well-encapsulated and tend to push the adjacent brain parenchyma, rather than infiltrate. During this process, tumor cells alter the surrounding ECM components, therefore, affecting tissue biomechanical properties (Marta et al., 2011).

Glioblastoma multiforme (GBM), a subtype of astrocytoma, is the most common and most aggressive brain tumor type. These tumors are extremely invasive due to their ability to remodel the surrounding ECM, through mechanisms that seem to involve integrin upregulation, matrix metalloproteinase (MMP) mediated proteolysis and *de novo* secretion of ECM proteins by malignant cells (Demuth and Berens, 2004). Moreover, cultured GBM cellular activities, such as proliferation, motility and mechanics were shown to be highly sensitive to changes in ECM stiffness (Sen et al., 2009; Ulrich et al., 2009). Notably, the constitutive activation of myosin-dependent contractility sensitized glioma tumor-initiating cells to mechanical inputs and reduced tissue invasion (Wong et al., 2015). The design of a bioengineered 3D poly(ethylene-glycol) (PEG)-based hydrogel tumor model contributed to elucidate the effects of matrix stiffness on glioblastoma cell behavior (Wang et al., 2014). Accordingly, modifications on matrix stiffness modulated cell proliferation, morphology and migration. Cells cultured on stiff hydrogels showed upregulation of hyaluronan synthase 1 and MMP1. This indicates that alterations in tensional homeostasis may play a role in cancer progression. Indeed, mechanical properties of the brain microenvironment were shown to alter glioblastoma cell migration *in vitro* and *in vivo* (Hegedus et al., 2006; Grundy et al., 2016), the expression of contractility-mediating signaling molecules, including RhoA and RhoB, and to correlate with tumor malignancy (Forget et al., 2002). In this context, RhoB might regulate cell cycle arrest, apoptosis through p53 activation (Ma et al., 2015). Further discussion on the signaling pathways involved in this pathology will be addressed in section “The Extracellular Matrix Role in the Process of Mechanotransduction.”

In all these CNS malignancies, individual cells remodel and diffusely invade the surrounding ECM, affecting tissue stiffness (Chauvet et al., 2016). In fact, tissue stiffness has been already exploited to detect cancer, using MRE and sono-elastography (Pepin et al., 2015; Chauvet et al., 2016). In tumors outside the CNS, the alterations in stiffness have been related with cellular malignancy and metastatic potential (Swaminathan et al., 2011). Qazi et al. (2011) have suggested that the differential invasive potential of tumors may be explained by mechanotransduction forces, and that fluid shear stress may lower glioma cell motility through modulation of MMPs activity. Nevertheless, MMP inhibitors have failed in clinical trials with patients with several types of cancer, including glioblastoma (Coussens et al., 2002). Moreover, some preclinical data suggests that MMP inhibition stimulates disease progression (Cathcart et al., 2015), as MMPs can degrade the ECM, making it less dense, enabling tumor invasion and proliferation. Focal adhesions have emerged as targets of interest for glioblastoma due to their central role in transducing mechanical signals between the cytoskeleton and the external ECM components (Sen et al., 2012). Taking this in consideration, Sen and coworkers showed that alpha-actinin isoforms participate in mechano-mechanical feedback between glioma cells and the ECM (Sen et al., 2009). Later, talin-1 was identified as a focal adhesion protein crucial for regulation of glioma cell spreading, motility and adaptation to ECM stiffness (Sen et al., 2012). These hypotheses need to be further validated in a 3D environment that presents a more complex combination of mechanical and topological cues.

As in other pathological conditions it is still unknown whether altered ECM mechanical properties play a key role in the establishment of brain tumors, but it is clear that it plays a vital role in tumor progression.

## Mechanotransduction and Central Nervous System Regeneration

Ramón y Cajal has stated that the CNS regenerative capacity is limited (Ramón, 1928) and still today, many decades later, this statement prevails. As previously discussed, alterations in CNS cells’ mechanical microenvironment may be the trigger to pathological states or part of the response to an insult. If on one hand CNS regeneration can be frustrated due to the glial scar, on the other hand, its success is believed to be dependent on physical cues.

Weiss (1934) acquired early evidences that topographical features of the substrate might guide axons. More specifically, they noticed that the trajectory of axons aligned parallel to grooves generated by brushed clotting blood onto a glass coverslip. Recent studies have clarified the role of several physical parameters involved in the regenerative processes of CNS cells by carefully engineering substrates able to vary rigidity and topography, while maintaining a constant chemical composition. Several authors have addressed the influence of mechanical properties on neural stem cell (NSC) differentiation, with potential impact at the NSC niche level or in regenerative cell-based therapies based on these cells. These studies suggest a direct role of stiffness either on the regulation of NSC lineage commitment (Saha et al., 2008; Banerjee et al., 2009) as NSC can generate the three main cell types of the CNS (neurons, oligodendrocytes and astrocytes), or on differently favoring cell survival (Saha et al., 2008). Regarding neuronal differentiation most of the studies had their focus on finding optimal neuronal differentiation stiffness conditions. The determined values were found to be within the elastic modulus of native brain tissue (*E* = 100–1,000 Pa) (Leipzig and Shoichet, 2009). A study with CNS cell lines, using photocrosslinkable methacrylate-chitosan hydrogels with incorporated laminin, showed different optimum stiffness values for NSC proliferation (3.5 kPa) and neuronal differentiation (< 1 kPa) (Leipzig and Shoichet, 2009). Interestingly, oligodendrocyte and astrocyte differentiation ability were favored in stiffer substrates (7 and 3.5 kPa, respectively) and were closely related to neural proliferation rates, as well as the presence of neurons. Although a myelinating population was found in all tested stiffness, maturation differences were seen among conditions: higher expression of myelin oligodendrocyte glycoprotein (MOG) was observed in substrates with stiffness values lower than 1 kPa, where more neurons were present. Concerning astrocytic differentiation, contradictory results can be found in the literature. Jiang et al. (2007) investigated the cellular response to substrate compliance using polyacrylamide gels, suggesting an optimal range of stiffness values for immature astrocytes (vimentin but not GFAP expressing cells), and their proliferative or differentiating status. These authors state that astrocytes preferentially adhered to stiffer substrates (>300 Pa). On the other hand, the response of mature astrocytes (GFAP expressing cells) was different, with higher cell adhesion, being observed on gels of intermediate stiffness. Another study (Moshayedi et al., 2010) has evidenced that mature astrocytes adhere to both soft (100 Pa) and stiff (10 kPa) polyacrylamide gels but with morphological differences, which is relevant in CNS disorder scenarios where tissue mechanical properties are altered. These studies report that astrocytes spread more and acquire more complex morphologies on stiff substrates, as observed on tissue culture polystyrene and glass. Nevertheless, astrocytes cultured on soft substrates resembled the star-like shape of astrocytes *in vivo*. We have also correlated substrate stiffness with astrocyte reactivity (Rocha et al., 2015). Astrocytes cultured in 3D alginate-based hydrogels acquired a reactive phenotype when compared to gels of intermediate rigidity. More recently, others showed that astrocytes cultured in poly(dimethyl siloxane) (PDMS) substrates with stiffness resembling healthy CNS (∼200 Pa) acquired a quiescent phenotype while in stiffer substrates (∼8,000 Pa) an astrogliosis-like phenotype was observed (Wilson et al., 2016).

The relationship between astrocytes and neurons is known to be important *in vivo*, though for many years astrocytes’ main function was believed to serve simply as an inert support for neurons. Lu et al. (2006) have studied the viscoelastic properties of CNS individual glial cells and neurons. The authors have analyzed intact rodent and bovine hippocampal and retina tissue samples using a scanning force microscope and a rheometer. CNS tissues were found to display the rheological characteristics of elastic solids. Moreover, astrocytes were determined to be about twice as soft as neurons, suggesting that glial cells act as soft compliant substrates surrounding neurons, instead of a rigid scaffold, which mechanically supports them. This implies a glial cell role closer to the original idea of Rudolph Virchow, who considered it to act as “brain glue” (Virchow, 1858). Glial cells have further shown a viscoelastic behavior, much like shock absorbers, when subjected to deformation. Interestingly, this data is in line with reports showing that neuron outgrowth is favored by soft substrates (Jiang et al., 2007; Rocha et al., 2014; Lantoine et al., 2016). Also in agreement with these results, Georges et al. (2006) and Jiang et al. (2007) have demonstrated that, while in the presence of astrocytes, neuron growth was independent of the substrate (polyacrylamide gel) stiffness. The same behavior was not seen when neurons were cultured alone, being able to extend neurites only on soft substrates. Therefore, a hard substrate may not constitute a problem for neuronal growth, as long as it is covered with a “cushioning” astrocytic layer. Notably, cells sense the microenvironment nanomechanical properties very closely and respond to them in a very effective way (Rocha et al., 2014). As such, neurons were able to sense the astrocytic layer, overcoming the impact of the stiff substrate underneath. This highlights surface nanomechanical properties importance in relation to the bulk properties.

During development, after neurons have reached their final destination and astrocytes have formed a 3D surrounding network, OPCs migrate and start to establish contact with axons. Myelination and its regenerative counterpart remyelination represent one of the most complex cell–cell interactions in the CNS (Jagielska et al., 2012). Kippert et al. (2009) have shown that a particular balance of matrix rigidity and intracellular contractile forces, mediated by the oligodendrocyte actomyosin cytoskeleton, is required for successful myelination and remyelination to occur. Moreover, Jagielska et al. (2012) evidenced that OPCs can differentiate on a wide range of substrate stiffness (0.1–70 kPa), which includes the range of stiffness found in the human brain (0.1–1 kPa). Furthermore, Leipzig and Schoichet (Leipzig and Shoichet, 2009) have observed that maturation and myelination ability were higher at < 1 kPa gels, while OPC survival was found to be optimum between 0.7–1 kPa. This is consistent with the fact that during development, OPCs migrate over a dense network of neurons and glia, being subjected to a broad range of mechanical forces (Gibson et al., 2014; Carvalho et al., 2022).

**FIGURE 3:**
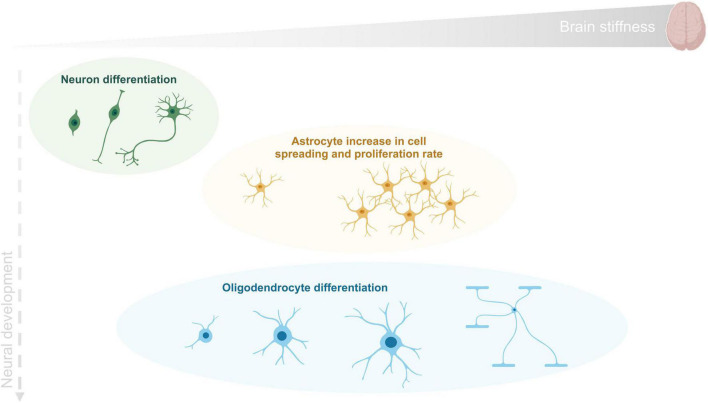
CNS cell differentiation during development as a function of tissue stiffness. Human brain reported stiffness ranges between 0.1 and 1 kPa. Neurons (in green) are generated first at the embryonic stage and differentiate in a softer environment than the remaining cells. Astrocytes (in orange) grow mainly on an environment with intermediate stiffness and finally oligodendrocytes (in blue) can proliferate and differentiate within the wide range of stiffness found in brain tissue.

Altogether these studies suggest that as for other tissues, CNS cellular morphology is determined by a precisely regulated interplay of intracellular contractile forces and extracellular attachment. Neurons, which are generated first at the embryonic stage, prefer relatively soft surfaces for elaboration and branching of axons and dendrites. These softer substrates possibly correspond to the environmental conditions at the time of initial path-finding of neuronal processes (Bauer and Ffrench-Constant, 2009). Astrocytes grow on intermediate substrates while, myelin-forming oligodendrocytes that develop later in the newborn stage, differentiate best on more rigid surfaces. In Figure 3, a schematic summary of the information here discussed is presented. Particular attention is given to the mechanical properties of the tissue and to their impact on cell behavior.

Notably, microglial cells, key players in CNS immune response, were shown to be also influenced by substrate features. Bollmann et al. (2015) have shown that although microglial cells preferentially migrate toward stiffer substrates, they have the ability to adapt their area, morphology and cytoskeleton according to the stiffness of the surrounding environment. Additionally, we have shown that microglial cells presented a round shape when cultured on flat substrates, while cells cultured on fibrous substrates exhibited elongated processes, responding with altered cytokine profile and myelin phagocytic capacity to these physical cues (Pires et al., 2015).

Besides stiffness, neural differentiation has been recently proved to be affected by other types of mechanical stimuli, including mechanical stress and shear forces. Arulmoli et al. (2015) investigated the effects of static stretch on neural cells differentiation. The authors showed that oligodendrocyte differentiation was impaired, contrarily to neuronal and astrocytic differentiation, which was not affected. This effect was dependent on ECM proteins (Arulmoli et al., 2015). Contrarily, Jagielska et al. (2017) showed that oligodendrocyte differentiation was increased with 10–15% static tensile strains but proliferation was inhibited. The authors proved that the most affected mechanotransduction related pathways were integrin, Rho-GTPases and actin cytoskeleton signaling.

Table 2 summarizes the response of each brain population to different mechanical stimuli (stiffness, topography and shear forces).

**TABLE 2:** The role of the different brain population to mechanical stimuli.

CNS cell population	Mechanical cue			References
	Topography	Stiffness	Shear forces	
NSC	Topographical cues along with protein interactions determine the migration and morphology of NSCs	Stiff: OPC and astrocytic differentiation favored Soft: neuron differentiation favored	Static stretch induces differentiation toward neurons and astrocytes	Saha et al., 2008; Banerjee et al., 2009; Leipzig and Shoichet, 2009; Arulmoli et al., 2015; Czeisler et al., 2016
Neurons	Align their trajectories with topographic cues	Soft: favors neuron differentiation	–	Georges et al., 2006; Jiang et al., 2007; Rocha et al., 2014; Lantoine et al., 2016
Astrocytes	Nanofibers ameliorate astrogliosis	Stiff: best adhesion Intermediate stiffness: spread more, complex morphologies (astrogliosis like)	Static stretch induces astrocytic differentiation	Jiang et al., 2007; Moshayedi et al., 2010; Min et al., 2013; Rocha et al., 2015; Wilson et al., 2016
Oligodendrocytes	OPCs align along microstructured platforms	Differentiation occurs within a wide range of stiffnesses (0.1–70 kPa) OPC survival: 0.7–1 kPa OPC maturation and myelination: < 1 kPa	Static stretch induces OPC differentiation	Webb et al., 1995; Bauer and Ffrench-Constant, 2009; Kippert et al., 2009; Leipzig and Shoichet, 2009; Jagielska et al., 2012, 2017; Gibson et al., 2014
Microglia	Fibrous substrates: microglia with elongated processes Flat substrates: round shaped microglia	Migration toward stiff substrates	–	Bollmann et al., 2015; Pires et al., 2015

*CNS, central nervous system; NSC, neural stem cell; OPC, oligodendrocyte precursor cell.*

## The Extracellular Matrix Role in the Process of Mechanotransduction

The ECM represents the secreted products of the CNS resident cells, serving a variety of cellular functions regulating synaptic transmission and plasticity, and constituting a barrier against metastatic invasive cancer cells (Crapo et al., 2012). Initially considered as an inert meshwork providing just physical support to cells, it has now been demonstrated its role as an active cell signaling modulator, working as a reservoir of enzymes, growth factors and of immunomodulatory cytokines and chemokines. Despite comprising only 20% of the brain volume, the architecture of the ECM may direct cell fate by providing structural and mechanical cues, which can affect cell transcriptional events and modulate cell phenotype and functions. ECM is known to influence cell differentiation, proliferation, survival, migration and invasion by both biochemical interactions (directly through cell adhesion, indirectly through presentation of arrested signaling molecules) and mechanical cues (stiffness, deformability). It is thought to play a vital role in maintenance of the normal tissue microenvironment and its misregulation leads to pathological conditions such as fibrosis, neuroinflammation, demyelination and cancer invasion (Raghow, 1994; Rosenberg, 2009; Klein and Bischoff, 2011).

Except for the meninges, vasculature and BBB, the ECM in the CNS has particular features as the proportion of fibrillar collagens and fibronectin is different than the one typically found in other tissues ECM. The CNS ECM is richer in adhesive glycoproteins (as tenascins or laminins) and proteoglycans (as lectican members). Some matrix components such as fibronectin, collagen type IV (non-fibrillar), and CSPGs are prominent and of known relevance to CNS plasticity and repair (Burnside and Bradbury, 2014). On the other hand, the presence of fibrillar collagen (collagen type I) is scarce, contributing to the softness of this tissue. For details on brain ECM biophysical, structural and chemical composition please refer to Chighizola et al. (2019).

As previously referred, the CNS ECM is extensively remodeled after a lesion. In traumatic injuries, astrogliosis and the glial scar formation induces a prominent matrix alteration with oversecretion of proteins as vimentin, collagen type IV, CSPGs and MMPs. Additionally, in demyelinating diseases the scenario is similar, with accumulation of laminin, hyaluronic acid and MMP-19 in MS plaques (Bonneh-Barkay and Wiley, 2009). The ECM changes throughout CNS pathologies have undoubtedly consequences in terms of biophysical properties of the environment, whose changes can be dramatic. Therefore, the role of ECM components is crucial for the development of chronic injuries and their expression can even dictate the outcome of degeneration/regeneration. It is also believed that targeting the ECM components that are over or underrepresented in a disease situation might be an interesting approach to treat neurodegeneration and a step closer to regeneration. For instance, as previously referred, CSPGs can be digested with chABC leading to restoration of function. Moreover, other ECM components can act as targets for promoting regeneration (such as heparin sulfate proteoglycans, hyaluronic acid, etc.) (Sun et al., 2021).

Nevertheless, the role of ECM components on mechanotransduction in this system is still poorly understood. Considering the large number of signaling receptors and mechanosensory motifs found in CNS cell surfaces, there are several potential pathways and engineering paradigms by which ECM mechanical signals could be transduced into biochemical signals (Vogel and Sheetz, 2006). Among these, integrins have been considered as the most plausible mechanosensor candidates, since they physically connect the ECM and the cytoskeleton, while acting as a signal transducer across cell membranes (Martin et al., 2002).

### Integrins

**FIGURE 4:**
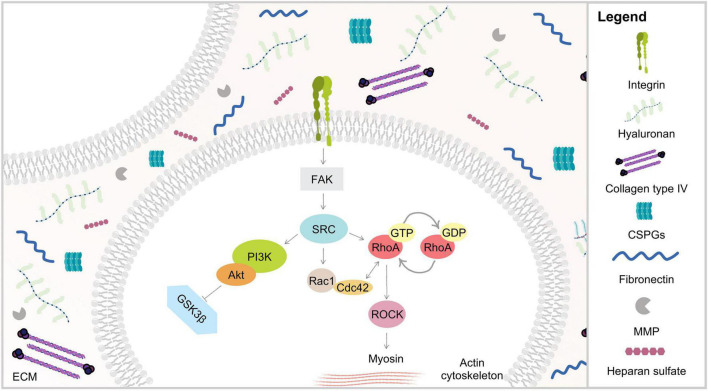
Schematic representation of ECM biophysical dynamics *via* integrins. Mechanical stimuli are mediated through ECM proteins binding to transmembrane integrin receptors, triggering several signaling cascades such as PI3K and Rho/ROCK. RhoA plays an important role in actin stress fibers assembly through its major downstream effector ROCK, known to promote myosin light chain phosphorylation and increasing cell contractility.

A growing body of evidence now suggests that the essential link between the mechanical properties of the extracellular environment and cellular decision-making are mechanotransductory processes at integrin-based cell-matrix contacts (Figure 4). In agreement, generated forces concentrate at cell-ECM adhesion and at cell–cell adhesion sites. Consequently, mechanotransduction is thought to occur within the multi-protein complexes of these adhesion sites. Mammalian cells usually co-express several integrins, which recognize distinct ECM components by binding specific amino-acid residues, such as the Arg-Gly-Asp (RGD) motif present in fibronectin, laminin or vitronectin (Humphries et al., 2006).

At the cell surface, integrins sense and respond to variations in force transmission in order to adapt cell-matrix adhesion to the ECM composition and properties (Geiger et al., 2009). The formation of ECM-integrin complexes promotes the integrin cytoplasmic domain to interact with the actin cytoskeleton and with other focal adhesion proteins, such as paxillin, talin and vinculin, as well as the formation of stress fibers (Mitra et al., 2005). ECM components, which interact with the extracellular domain of integrin receptors, are known to trigger, upon mechanical stimuli, a number of intracellular signaling cascades initiated by the phosphorylation of the focal adhesion kinase (FAK) (Geiger et al., 2009). The intimate contact between proteins of these adhesive structures and proteins of the actin/tubulin network induces extensive alterations at the cytoskeleton organization, leading to pronounced cell morphological modifications (Han and de Rooij, 2016). It has been observed that the application of force, using latex beads, on integrin complexes reinforced adhesive sites and mechanotransduction (Choquet et al., 1997). Later it has been shown that the force exerted externally by a micropipette leads to growth of those focal adhesions which are tensed (Riveline et al., 2001; Schiller et al., 2013). There is some evidence that other adhesion structures, such as podosomes and fibrillar adhesions, which also connect ECM proteins to the actin cytoskeleton, can also participate in mechanotransduction (Collin et al., 2008). However, still little is known about their role in mechanotransduction pathways.

FAK expression has been described in neurons (Grant et al., 1995; Contestabile et al., 2003), in oligodendrocytes (Kilpatrick et al., 2000; Bacon et al., 2007) and more recently in microglia (Choi et al., 2015; Kemmerling et al., 2017). FAK activity is known to be required for early events of cell adhesion in neuronal growth cones (Navarro and Rico, 2014), which sense guidance cues, and mechanically pull the axon forward. During this process, novel adhesion complexes are established at the cell migratory rear, while the cell cytoskeleton restructures leading to cell body contraction, old adhesion complexes are lost and the cell moves forward, establishing novel ECM adhesion contacts at the front (Yamaguchi et al., 2005). Therefore, FAK is simultaneously implicated in both adhesion complexes assembly and disassembly. Similarly, the guidance of growth cones is a multistep process that involves adhesion, assembly and disassembly. Moore et al. (2012) have studied the physical interactions of growth cones with their guidance cues. The authors have demonstrated the need for mechanical forces during chemo-attraction to netrin-1 and for the regulation of FAK and Crk-associated substrates. In fact, in axon guidance, FAK has also been previously implicated to a growing number of other cues, including: ephrins, semaphorins, and brain-derived neurotrophic factor (Falk et al., 2005; Woo and Gomez, 2006). As such, FAK may function as a mechanosensor in response to these guidance cues.

Oligodendrocytes also undergo important morphological changes through their maturation process. FAK as a key player in regulating cytoskeleton organization is also involved in the maturation and myelination process of oligodendrocytes. Indeed, Hoshina et al. (2007) reported that FAK mediates process outgrowth in an oligodendrocyte rat-derived cell line. Several other studies point to the idea that FAK is crucial for oligodendrocytes, particularly during the maturation process (Fox et al., 2004; Miyamoto et al., 2007; Rajasekharan et al., 2009). Moreover, Forrest and coworkers (Forrest et al., 2009) have shown that FAK conditional knock-out mice have some inhibition or delay of normal myelination during development, reinforcing the idea of a pivotal role of FAK during oligodendrocyte maturation.

Microglia cells, which are highly motile as they migrate toward lesion site after injury, are known to secrete many inflammatory cytokines. A recent study has shown that CSPG4 (also known as neuron-glial antigen 2, NG2) expressed by microglia during CNS injury leads to profound alterations in the morphology and function of these cells as well as in the secretion of inflammatory cytokines *via* FAK phosphorylation (Zhu et al., 2016). Moreover, microglia motility and migration have been proved to be closely related with FAK pathways being highly activated in the context of CNS injuries (Choi et al., 2015; Kemmerling et al., 2017).

### Rho/ROCK Signaling Pathway

It has become noticeable that there is a crosstalk between integrins and GTPases, as a consequence of the adhesion signaling cascades triggered during integrin sensing of ECM mechanical properties. In fact, the activation of integrins by mechanical forces is thought to result in the recruitment of intracellular mediators that signal through the Rho/Rho-associated coiled-coil-containing protein kinase (ROCK) signaling pathway to activate force-generating myosin II (Figure 4; Bauer and Ffrench-Constant, 2009). As such, the Rho family guanosine-5-triphosphatases (GTPases) are also crucial in mechanosensing of matrix stiffness, cell morphological alterations, and cytoskeleton tension (Keung et al., 2011). Moreover, it has been proposed that these signaling molecules may work together as a complex network, depending on their subcellular location or the cell cycle status (Hoffman et al., 2011). This complex network could provide cells with the necessary structure and flexibility to tune their responses under distinct physiological conditions.

The small GTPases Ras superfamily comprises 20 members, which are widely expressed in mammals, including RhoA, Rac1, and Cdc42. RhoA, the best-characterized of the Ras proteins, acts as a molecular switch, cycling between an inactive GDP-bound and an active GTP-bound conformation. This molecule has been widely implicated in integrin-mediated signaling, regulation of the assembly and organization of the actin cytoskeleton, and cell migration modulation (Etienne-Manneville and Hall, 2002). RhoA plays a critical role in the assembly of actin stress fibers in response to distinct soluble stimuli, including serum, growth factors, and lysophosphatidic acid (LPA), and to insoluble adhesion ligands such as fibronectin or other ECM components (Ren et al., 1999). It is also known to play a critical role in the assembly of actin stress fibers in response to applied mechanical forces (Putnam et al., 2003). Moreover, RhoA signaling was found to be closely associated to the pathogenesis of several nervous system disorders (Guan et al., 2013), and involved in many aspects of neuronal functions including neurite outgrowth and retraction (Tan et al., 2011). RhoA is a key regulator of intracellular contractility and, thus, allows cells to sense matrix stiffness and to respond to mechanical cues. This function is largely put forth through the RhoA major downstream effector, ROCK. ROCK is involved in regulating neural cell migration, proliferation, survival, communication, axon guidance, and regeneration (González-Forero et al., 2012; Maldonado et al., 2017). Wozniak and coworkers (Wozniak et al., 2003) demonstrated that when ROCK or RhoA activity are altered, cells no longer respond effectively to mechanical forces induced by increased matrix stiffness. Some authors have suggested that stiff matrices, in general, lead to increased Rho activation (Wozniak et al., 2003; Provenzano et al., 2009), whereas Rac1 activity remained unchanged, implicating that ECM stiffness preferentially activates specific Rho GTPases and consequently the formation of actin stress fibers (Keung et al., 2011). ROCK was found to be involved in focal adhesion formation by promoting myosin light chain (MLC) phosphorylation (Kimura et al., 1996), as well as increasing cell contractility (Chrzanowska-Wodnicka and Burridge, 1996; Matthews et al., 2006), promoting cell migration, polarization and differentiation (Wozniak et al., 2003). Several studies have confirmed that Rho and their associated signaling molecules actively mediate crucial neuron biological processes such as axon regeneration (Sivasankaran et al., 2004; Duffy et al., 2009), and have also been correlated with enhanced BBB permeability (Krizbai and Deli, 2003). Interestingly, Rho has also been correlated with increased cell proliferation in many cancers (Faried et al., 2007; Orgaz et al., 2014). Some studies provided additional evidences that ROCK inhibitors had potential therapeutic application for Alzheimer’s disease (Raad et al., 2012; Chen et al., 2017), Parkinson’s disease (Tonges et al., 2012; Roser et al., 2017), amyotrophic lateral sclerosis (ALS) (Roser et al., 2017), autoimmune neuritis (Pineda et al., 2011) and spinal cord injury (Pires et al., 2017). Given the established role for RhoA-ROCK-mediated cytoskeletal tension, not only in cell migration but also for cell proliferation, these pathways may play, an essential role in regulating both tissue homeostasis and malignant transformation.

### YAP/TAZ Signaling Pathway

Integrin binding leads to downstream signaling pathways which in turn regulate mechanosensors at the level of the nuclei (transcription factors). The YAP and its homologous protein TAZ have been a hot topic in the mechanobiology field. Briefly, these proteins shuttle between the cytoplasm and the nucleus depending on cell density, stretch, or stiffness cues. YAP and TAZ act as activators of transcription factors regulating genes involved in anti-apoptotic and proliferative pathways. When cells sense a stiff matrix or change their phenotype from a round shape to a stretched one, YAP/TAZ become activated (Cobbaut et al., 2020).

Apart from being activated by integrin receptors there are other mechanosensory channels that can interact with YAP and TAZ. For instance, Piezo-1 is the most well studied calcium-dependent channel that directly activates the nuclear translocation of YAP and TAZ (Wang et al., 2020). Transient potential receptor (TRP) channels, mechanosensors transmitters of pain and stretch stimuli, are also associated with YAP/TAZ activation (Van den Eynde et al., 2021).

Concerning this signaling pathway role in CNS homeostasis and pathology, it controls the balance between cell proliferation and apoptosis and is implied in the regulation of the volume of injured tissues. Specifically, YAP/TAZ has been shown to regulate the proliferation, differentiation and apoptosis of NSC (Luo and Li, 2022). In glioblastoma, the tumor nature of proliferative cells implies an upregulation of the YAP/TAZ signaling pathway (Jin et al., 2020). Additionally, the YAP/TAZ pathway has been demonstrated to be an important regulator of Alzheimer’s disease as it induces apoptosis and intracellular amyloid-beta mediated necrosis (Tanaka et al., 2020).

For further reading on CNS homeostasis and pathologies regulation by YAP/TAZ please refer to Jin et al. (2020).

### Matrix Metalloproteinases and Extracellular Matrix Remodeling

The ECM has remodeling enzymes, MMPs, which are a large family of proteases involved in many cell-matrix and cell-cell signaling processes. Mammalian MMPs share a conserved domain that consists of a catalytic and an autoinhibitory pro-domain (Page-McCaw et al., 2007). Collectively they can cleave all protein components of the ECM, as well as other substrates including growth factors, cell adhesion molecules and receptors (McCawley and Matrisian, 2001). MMP proteolysis is now known to create space for cells to migrate, to produce specific substrate-cleavage fragments which become biologically active, to regulate tissue architecture and influence the activity of signaling molecules, both directly and indirectly (Sternlicht and Werb, 2001).

Uncontrolled MMP activity underlies the pathophysiology of many disorders, such as cancer, asthma, rheumatoid arthritis and retinal detachment (Sivak and Fini, 2002; Imamura et al., 2008; Martins-Oliveira et al., 2009), and has also been associated with neurodegenerative diseases like glaucoma, migraine, Alzheimer’s disease, Parkinson’s disease, amyotrophic lateral sclerosis and MS (Rosario Hernandez and Pena, 1997; Yong, 2005; Rosenberg, 2009; Klein and Bischoff, 2011). Although high levels of MMPs often correlate with poor prognosis in human patients (Egeblad and Werb, 2002), their up-regulation is believed to underlie reparative functions in the CNS at well-defined places and time points after an insult (Yong, 2005).

Given the presence of receptors, like integrins, for ECM components on cells, and the ability of MMPs to cleave virtually all ECM components, these enzymes may influence cellular function by regulating the ECM composition and concomitantly have a crucial role on ECM mechanical properties, playing a part in mechanotransduction events. Although this relationship has not been widely studied, particularly in the CNS, some evidences were found in other systems. In the uterine cervix it has been shown that MMPs contribute to de-stiffening which precedes and facilitates the dilatation of the cervix during fetal delivery (Rahkonen et al., 2009). MMPs were also shown to modulate the mechanical properties of the compass depressor ligaments of echinoderms (Ribeiro et al., 2012) and have also been documented to be involved in osteocyte response to mechanical loading (Kulkarni et al., 2012).

Moreover, it has been described that in neuronal injury scenarios MMP levels are increased (Mroczko et al., 2013). This observation, combined with the altered stiffness of the injured CNS tissue in relation to the healthy tissue, suggest that increased MMP levels at the injury site lead to increased re-organization of the ECM, with a resulting alteration of the cell cytoskeleton network and mechanical properties.

## Exploring Mechanotransduction in the Context of Central Nervous System Diseases: Current Clinical Strategies and Future Perspectives

Physical cues are important regulators of several biological functions during distinct momentums of cellular life. The disease contexts explored in this review demonstrate that the processes of mechanotransduction are important contributors to the alteration of tissue function. Nonetheless, the interplay between mechanical induction of signaling pathways and disease is still a largely unexplored target for therapeutic intervention.

There are several reports relative to mechanosensing proteins, suggesting the existence of multiple mechanisms, though it is not known whether these are redundant or complementary. The future work to be performed in the field needs to address the molecular and biophysical basis of CNS mechanotransduction. This will require a multidisciplinary approach, with a combination of molecular biology and bioengineering techniques. For now, studies have been mainly focused on affecting one pathway, like knocking down or inhibiting one protein. Most certainly, in the near future, there will be the need for high throughput systems which enable the simultaneous analysis of distinct and interrelated pathways and the evaluation of the effects of combinatorial drugs and treatments. Although efforts have been made at the molecular level, we still need to increase our understanding of the dynamics of mechanotransduction in health and disease, beyond the existent knowledge on focal adhesions and ion channels. Therefore, there is a growing effort to study such issues at the tissue and organ level, and imaging techniques such as MRE will expectedly take us closer to future findings.

It must be highlighted that the interest on mechanotransduction, in the context of CNS pathologies has already evolved from bench to bedside, as seen by the increasing number of studies with MRE neuroimaging techniques. These aim at understanding the correlation of brain mechanical properties with function. In fact, MRE has been under clinical trials to assess its utility in the non-invasive diagnosis of glial tumors (NCT03274037) and idiopathic intracranial hypertension (NCT03096743).

The enhancement of CNS regeneration by targeting ECM molecules known to inhibit regeneration has also been under evaluation. A few strategies have been tested such as the use of chABC, Nogo-A inhibitors and RhoA inhibitors. These will ultimately target mechanotransduction processes.

chABC has been tested mainly in spinal cord injury scenarios, due to its ability to digest the scar tissue that prevents CNS regeneration after injury, with the final aim of enhancing plasticity and promoting effective rehabilitation. Several veterinary clinical trials were performed aiming at treating spinal cord injuries in dogs, namely at Iowa State University. Results from these studies are promising leading to improved walking and bladder functions (Hu et al., 2018). Recently, chABC has also been tested in rhesus monkeys with promising results both in terms of improved mobility and cellular regenerating mechanisms (increased axonal growth and synapse formation) (Rosenzweig et al., 2019). In fact, spinal cord injury (SCI) is one of the most common neurological pathologies in veterinary medicine due to the degeneration of intervertebral disks, which then rupture and cause injury to the spinal cord. Human clinical trials with chABC are now closer to become a reality as a modified enzyme is being investigated at the Cambridge Center for Brain Repair toward the development of a human safe formulation.

Antibodies against Nogo-A work through inhibition of Nogo, a myelin associated neurite outgrowth inhibitor which is known to activate the Rho-ROCK pathway. Although Nogo has been widely correlated with axonal growth, it may play important roles on other mechanisms and anti-NogoA drugs were recently under phase I clinical trials in patients with Multiple Sclerosis (NCT01424423 and NCT01435993).

Regarding Rho inhibition, two drugs have been currently under focus: ibuprofen and C3 transferase. Ibuprofen has been on the market as an anti-inflammatory drug for a long time, but new formulations and applications are currently under investigation. In fact, a phase I clinical trial to treat acute traumatic spinal cord injury (NCT02096913) was finished in 2017 but the results are still not available. It has also been explored for the treatment of mild traumatic brain injuries in Phase II clinical trials (NCT02443142). Nonetheless, there are no available public results. Additionally, the interest in ibuprofen has been expanding to be used as a therapeutic agent in Alzheimer’s disease as it was found to reduce Aβ42 levels by modulating the activity of the γ-secretase enzyme complex. Although the first clinical trials in patients with mild to moderate Alzheimer’s disease revealed no differences between the ibuprofen treated and placebo groups (Pasqualetti et al., 2009), a more recent study claimed that the early and continuous administration of ibuprofen can have a beneficial effect in preventing Alzheimer’s disease (McGeer et al., 2018).

C3 transferase has entered clinical trials PhaseI/II under the name Cethrin^®^ and results suggest an increase in neurological recovery after SCI. BioAxone BioSciences started a phase II/III trial for SCI in United States and Canada (NCT02053883) in 2014 with this molecule and Vertex Pharmaceuticals completed a PhaseI/II study with this same molecule for the treatment of acute thoracic and cervical spinal cord injuries (NCT00500812) in 2016. The same company just finished a Phase II/III clinical trial for the treatment of acute traumatic cervical spinal cord injury (NCT02669849) but the outcomes are yet to be published.

While a large number of issues related to the process of mechanotransduction remains to be addressed, particularly in the context of CNS, basic and clinical research of the last decades led to increasing understanding of this topic, putting it forward as a valuable diagnostic tool and therapeutic target. Undoubtedly an increasing number of therapies are currently seeking translation into human clinical trials. Nevertheless, one of the future challenges facing the biomedicine field will be the development of effective therapies, based on the advances achieved on basic research.

## Author Contributions

DR and AP conceived the general idea for the article. DR and EC performed literature research and designed the figures/tables. DR wrote the manuscript. EC provided input on the writing of sections “Mechanotransduction in CNS Pathology,” “Mechanotransduction and CNS Regeneration,” and “Exploring Mechanotransduction in the Context of CNS Diseases: Current Clinical Strategies and Future Perspectives.” JR and MO revised the work and gave input on sections “Cancer” and “Integrins,” respectively. AP supervised and critically reviewed the article, as well as assured the procurement of funding. All authors contributed to the article and approved the submitted version.

## Conflict of Interest

The authors declare that the research was conducted in the absence of any commercial or financial relationships that could be construed as a potential conflict of interest.

## Publisher’s Note

All claims expressed in this article are solely those of the authors and do not necessarily represent those of their affiliated organizations, or those of the publisher, the editors and the reviewers. Any product that may be evaluated in this article, or claim that may be made by its manufacturer, is not guaranteed or endorsed by the publisher.

## References

[B1] Agrawal N. J. Radhakrishnan R. (2009). Calculation of free energies in fluid membranes subject to heterogeneous curvature fields. *Phys. Rev.* 80:11925. 10.1103/PhysRevE.80.011925 19658747 PMC2803019

[B2] Alexander A. L. Lee J. E. Field A. S. (2007). Diffusion tensor imaging of the Brain. *Neurotherapeutics* 4 316–329. 17599699 10.1016/j.nurt.2007.05.011 PMC2041910

[B3] Arulmoli J. Pathak M. M. McDonnell L. P. Nourse J. L. Tombola F. Earthman J. C. (2015). Static stretch affects neural stem cell differentiation in an extracellular matrix-dependent manner. *Sci. Rep.* 5:8499. 10.1038/srep08499 25686615 PMC4330529

[B4] Back S. A. Tuohy T. M. Chen H. Wallingford N. Craig A. Struve J. (2005). Hyaluronan accumulates in demyelinated lesions and inhibits oligodendrocyte progenitor maturation. *Nat. Med.* 11 966–972. 10.1038/nm1279 16086023

[B5] Bacon C. Lakics V. Machesky L. Rumsby M. (2007). N-WASP regulates extension of filopodia and processes by oligodendrocyte progenitors, oligodendrocytes, and Schwann cells - Implications for axon ensheathment at myelination. *GLIA* 55 844–858. 10.1002/glia.20505 17405146

[B6] Bahra A. Matharu M. S. Buchel C. Frackowiak R. S. Goadsby P. J. (2001). Brainstem activation specific to migraine headache. *Lancet* 357 1016–1017. 10.1016/s0140-6736(00)04250-1 11293599

[B7] Banerjee A. Arha M. Choudhary S. Ashton R. S. Bhatia S. R. Schaffer D. V. (2009). The influence of hydrogel modulus on the proliferation and differentiation of encapsulated neural stem cells. *Biomaterials* 30 4695–4699. 10.1016/j.biomaterials.2009.05.050 19539367 PMC2743317

[B8] Bauer N. G. Ffrench-Constant C. (2009). Physical forces in myelination and repair: a question of balance? *J. Biol.* 8:78. 10.1186/jbiol169 19804608 PMC2776912

[B9] Bissell M. J. Hall H. G. Parry G. (1982). How does the extracellular matrix direct gene expression? *J. Theoret. Biol.* 99 31–68. 10.1016/0022-5193(82)90388-5 6892044

[B10] Bollmann L. Koser D. E. Shahapure R. Gautier H. O. Holzapfel G. A. Scarcelli G. (2015). Microglia mechanics: immune activation alters traction forces and durotaxis. *Front. Cell Neurosci.* 9:363. 10.3389/fncel.2015.00363 26441534 PMC4585148

[B11] Bonneh-Barkay D. Wiley C. A. (2009). Brain extracellular matrix in neurodegeneration. *Brain Pathol.* 19 573–585. 10.1111/j.1750-3639.2008.00195.x 18662234 PMC2742568

[B12] Bradbury E. J. Moon L. D. Popat R. J. King V. R. Bennett G. S. Patel P. N. (2002). Chondroitinase ABC promotes functional recovery after spinal cord injury. *Nature* 416 636–640. 10.1038/416636a 11948352

[B13] Buck L. Axel R. (1991). A novel multigene family may encode odorant receptors: a molecular basis for odor recognition. *Cell* 65 175–187. 10.1016/0092-8674(91)90418-x 1840504

[B14] Burnside E. R. Bradbury E. J. (2014). Review: manipulating the extracellular matrix and its role in brain and spinal cord plasticity and repair. *Neuropathol. Appl. Neurobiol.* 40 26–59. 10.1111/nan.12114 24438519

[B15] Caggiano A. O. Zimber M. P. Ganguly A. Blight A. R. Gruskin E. A. (2005). Chondroitinase ABCI improves locomotion and bladder function following contusion injury of the rat spinal cord. *J. Neurotrauma* 22 226–239. 10.1089/neu.2005.22.226 15716629

[B16] Carvalho E. D. Morais M. R. G. Ferreira H. P. Silva M. M. C. Guimarães S. C. Pêgo A. P. (2022). A paradigm shift: bioengineering meets mechanobiology towards overcoming remyelination failure. *Biomaterials* 2022:121427. 10.1016/j.biomaterials.2022.121427 35276617

[B17] Cathcart J. Pulkoski-Gross A. Cao J. (2015). Targeting matrix metalloproteinases in cancer: bringing new life to old ideas. *Genes Dis.* 2 26–34. 10.1016/j.gendis.2014.12.002 26097889 PMC4474140

[B18] Chauvet D. Imbault M. Capelle L. Demene C. Mossad M. Karachi C. (2016). In Vivo Measurement of Brain Tumor Elasticity Using Intraoperative Shear Wave Elastography. *Ultraschall Med.* 37 584–590. 10.1055/s-0034-1399152 25876221

[B19] Chen J. Sun Z. Jin M. Tu Y. Wang S. Yang X. (2017). Inhibition of AGEs/RAGE/Rho/ROCK pathway suppresses non-specific neuroinflammation by regulating BV2 microglial M1/M2 polarization through the NF-kappaB pathway. *J. Neuroimmunol.* 305 108–114. 10.1016/j.jneuroim.2017.02.010 28284330

[B20] Chighizola M. Dini T. Lenardi C. Milani P. Podestà A. Schulte C. (2019). Mechanotransduction in neuronal cell development and functioning. *Biophys. Rev.* 11 701–720. 10.1007/s12551-019-00587-2 31617079 PMC6815321

[B21] Choi I. Kim B. Byun J. W. Baik S. H. Huh Y. H. Kim J. H. (2015). LRRK2 G2019S mutation attenuates microglial motility by inhibiting focal adhesion kinase. *Nat. Commun.* 6:8255. 10.1038/ncomms9255 26365310 PMC4647842

[B22] Choquet D. Felsenfeld D. P. Sheetz M. P. (1997). Extracellular matrix rigidity causes strengthening of integrin- cytoskeleton linkages. *Cell* 88 39–48. 10.1016/s0092-8674(00)81856-5 9019403

[B23] Chou T. Siegel M. (2012). A mechanical model of retinal detachment. *Phys. Biol.* 9:46001. 10.1088/1478-3975/9/4/046001 22733081

[B24] Chrzanowska-Wodnicka M. Burridge K. (1996). Rho-stimulated contractility drives the formation of stress fibers and focal adhesions. *J. Cell Biol.* 133 1403–1415. 10.1083/jcb.133.6.1403 8682874 PMC2120895

[B25] Cobbaut M. Karagil S. Bruno L. Diaz de la Loza M. D. C. Mackenzie F. E. Stolinski M. (2020). Dysfunctional Mechanotransduction through the YAP/TAZ/Hippo Pathway as a Feature of Chronic Disease. *Cells* 9:cells9010151. 10.3390/cells9010151 31936297 PMC7016982

[B26] Collin O. Na S. Chowdhury F. Hong M. Shin M. E. Wang F. (2008). Self-organized podosomes are dynamic mechanosensors. *Curr. Biol.* 18 1288–1294. 10.1016/j.cub.2008.07.046 18760605 PMC2605691

[B27] Contestabile A. Bonanomi D. Burgaya F. Girault J. A. Valtorta F. (2003). Localization of focal adhesion kinase isoforms in cells of the central nervous system. *Int. J. Dev. Neurosci.* 21 83–93. 10.1016/s0736-5748(02)00126-0 12615084

[B28] Corey D. P. Hudspeth A. J. (1979). Response latency of vertebrate hair cells. *Biophys. J.* 26 499–506. 10.1016/S0006-3495(79)85267-4 318064 PMC1328566

[B29] Coussens L. M. Fingleton B. Matrisian L. M. (2002). Matrix metalloproteinase inhibitors and cancer: trials and tribulations. *Science* 295 2387–2392. 10.1126/science.1067100 11923519

[B30] Crapo P. M. Medberry C. J. Reing J. E. Tottey S. van der Merwe Y. Jones K. E. (2012). Biologic scaffolds composed of central nervous system extracellular matrix. *Biomaterials* 33 3539–3547. 10.1016/j.biomaterials.2012.01.044 22341938 PMC3516286

[B31] Czeisler C. Short A. Nelson T. Gygli P. Ortiz C. Catacutan F. P. (2016). Surface topography during neural stem cell differentiation regulates cell migration and cell morphology. *J. Comp. Neurol.* 524 3485–3502. 10.1002/cne.24078 27418162

[B32] Davies P. F. Barbee K. A. Volin M. V. Robotewskyj A. Chen J. Joseph L. (1997). Spatial relationships in early signaling events of flow-mediated endothelial mechanotransduction. *Annu. Rev. Physiol.* 59 527–549. 10.1146/annurev.physiol.59.1.527 9074776

[B33] Davies P. F. Robotewskyj A. Griem M. L. Dull R. O. Polacek D. C. (1992). Hemodynamic forces and vascular cell communication in arteries. *Arch. Pathol. Lab. Med.* 116 1301–1306. 1456875

[B34] Davis J. T. Wen Q. Janmey P. A. Otteson D. C. Foster W. J. (2012). Muller cell expression of genes implicated in proliferative vitreoretinopathy is influenced by substrate elastic modulus. *Investig. Ophthalmol. Vis. Sci.* 53 3014–3019. 10.1167/iovs.11-8450 22447866 PMC3378084

[B35] Delmas P. Hao J. Rodat-Despoix L. (2011). Molecular mechanisms of mechanotransduction in mammalian sensory neurons. *Nat. Rev. Neurosci.* 12 139–153. 10.1038/nrn2993 21304548

[B36] Demuth T. Berens M. E. (2004). Molecular mechanisms of glioma cell migration and invasion. *J. Neurooncol.* 70 217–228. 10.1007/s11060-004-2751-6 15674479

[B37] DeTure M. A. Dickson D. W. (2019). The neuropathological diagnosis of Alzheimer’s disease. *Mol. Neurodegen.* 14:32. 10.1186/s13024-019-0333-5 31375134 PMC6679484

[B38] Di Castro A. Drew L. J. Wood J. N. Cesare P. (2006). Modulation of sensory neuron mechanotransduction by PKC- and nerve growth factor-dependent pathways. *Proc. Natl. Acad. Sci. USA.* 103 4699–4704. 10.1073/pnas.0508005103 16537426 PMC1450234

[B39] Donat C. K. Scott G. Gentleman S. M. Sastre M. (2017). Microglial Activation in Traumatic Brain Injury. *Front. Aging Neurosci.* 9:208–208. 10.3389/fnagi.2017.00208 28701948 PMC5487478

[B40] Duffy P. Schmandke A. Sigworth J. Narumiya S. Cafferty W. B. Strittmatter S. M. (2009). Rho-associated kinase II (ROCKII) limits axonal growth after trauma within the adult mouse spinal cord. *J. Neurosci.* 29 15266–15276. 10.1523/JNEUROSCI.4650-09.2009 19955379 PMC2855556

[B41] Dupont S. Morsut L. Aragona M. Enzo E. Giulitti S. Cordenonsi M. (2011). Role of YAP/TAZ in mechanotransduction. *Nature* 474 179–184. 21654799 10.1038/nature10137

[B42] Eckert G. P. Cairns N. J. Maras A. Gattaz W. F. Muller W. E. (2000). Cholesterol modulates the membrane-disordering effects of beta-amyloid peptides in the hippocampus: specific changes in Alzheimer’s disease. *Dement. Geriatric Cognit. Disord.* 11 181–186. 10.1159/000017234 10867442

[B43] Edvinsson L. Uddman R. (2005). Neurobiology in primary headaches. *Brain Res. Rev.* 48 438–456. 10.1016/j.brainresrev.2004.09.007 15914251

[B44] Egeblad M. Werb Z. (2002). New functions for the matrix metalloproteinases in cancer progression. *Nat. Rev. Cancer* 2 161–174. 10.1038/nrc745 11990853

[B45] ElSheikh M. Arani A. Perry A. Boeve B. F. Meyer F. B. Savica R. (2017). MR Elastography Demonstrates Unique Regional Brain Stiffness Patterns in Dementias. *AJR Am. J. Roentgenol.* 209 403–408. 10.2214/ajr.16.17455 28570101 PMC5597304

[B46] Etienne-Manneville S. Hall A. (2002). Rho GTPases in cell biology. *Nature* 420 629–635. 12478284 10.1038/nature01148

[B47] Falk J. Bechara A. Fiore R. Nawabi H. Zhou H. Hoyo-Becerra C. (2005). Erratum: dual functional activity of semaphorin 3B is required for positioning the anterior commissure (Neuron (October 6, 2005) 48 (63-75). *Neuron* 48:699. 10.1016/j.neuron.2005.08.033 16202709

[B48] Faried A. Faried L. S. Usman N. Kato H. Kuwano H. (2007). Clinical and prognostic significance of RhoA and RhoC gene expression in esophageal squamous cell carcinoma. *Ann. Surg. Oncol.* 14 3593–3601. 10.1245/s10434-007-9562-x 17896152

[B49] Fawcett J. W. Asher R. A. (1999). The glial scar and central nervous system repair. *Brain Res. Bull.* 49 377–391. 10.1016/s0361-9230(99)00072-6 10483914

[B50] Ferrari M. D. (1998). Migraine. *Lancet* 351 1043–1051. 10.1016/S0140-6736(97)11370-8 9546526

[B51] Finocchi C. Sassos D. (2017). Headache and arterial hypertension. *Neurol. Sci.* 38 (Suppl. 1), 67–72. 10.1007/s10072-017-2893-x 28527058

[B52] Forget M. A. Desrosiers R. R. Del M. Moumdjian R. Shedid D. Berthelet F. (2002). The expression of rho proteins decreases with human brain tumor progression: potential tumor markers. *Clin. Exp. Metastasis* 19 9–15. 10.1023/a:1013884426692 11918088

[B53] Forrest A. D. Beggs H. E. Reichardt L. F. Dupree J. L. Colello R. J. Fuss B. (2009). Focal Adhesion Kinase (FAK): a regulator of CNS myelination. *J. Neurosci. Res.* 87 3456–3464. 10.1002/jnr.22022 19224576 PMC2760606

[B54] Fox M. A. Alexander J. K. Afshari F. S. Collelo R. J. Fuss B. (2004). Phosphodiesterase-I alpha/autotaxin controls cytoskeletal organization and FAK phosphorylation during myelination. *Mol. Cell Neurosci.* 27 140–150. 10.1016/j.mcn.2004.06.002 15485770

[B55] Freimann F. B. Streitberger K. J. Klatt D. Lin K. McLaughlin J. Braun J. (2011). Alteration of brain viscoelasticity after shunt treatment in normal pressure hydrocephalus. *Neuroradiology* 54 189–196. 10.1007/s00234-011-0871-1 21538046

[B56] Gardener H. Monteith T. Rundek T. Wright C. B. Elkind M. S. V. Sacco R. L. (2016). Hypertension and Migraine in the Northern Manhattan Study. *Ethnicity Dis.* 26 323–330. 10.18865/ed.26.3.323 27440971 PMC4948798

[B57] Geiger B. Spatz J. P. Bershadsky A. D. (2009). Environmental sensing through focal adhesions. *Nat. Rev. Mol. Cell Biol.* 10 21–33. 10.1038/nrm2593 19197329

[B58] Georges P. C. Miller W. J. Meaney D. F. Sawyer E. S. Janmey P. A. (2006). Matrices with compliance comparable to that of brain tissue select neuronal over glial growth in mixed cortical cultures. *Biophys. J.* 90 3012–3018. 10.1529/biophysj.105.073114 16461391 PMC1414567

[B59] Gerischer L. M. Fehlner A. Köbe T. Prehn K. Antonenko D. Grittner U. (2018). Combining viscoelasticity, diffusivity and volume of the hippocampus for the diagnosis of Alzheimer’s disease based on magnetic resonance imaging. *NeuroImage Clin.* 18 485–493. 10.1016/j.nicl.2017.12.023 29527504 PMC5842309

[B60] Gibson E. M. Purger D. Mount C. W. Goldstein A. K. Lin G. L. Wood L. S. (2014). Neuronal activity promotes oligodendrogenesis and adaptive myelination in the mammalian brain. *Science* 344:1252304. 10.1126/science.1252304 24727982 PMC4096908

[B61] Goadsby P.J. Edvinsson L. Ekman R. (1990). Vasoactive peptide release in the extracerebral circulation of humans during migraine headache. *Ann. Neurol.* 28, 183–187. 10.1002/ana.410280213 1699472

[B62] González-Forero D. Montero F. Garcia-Morales V. Domínguez G. Gómez-Pérez L. García-Verdugo J. M. (2012). Endogenous rho-kinase signaling maintains synaptic strength by stabilizing the size of the readily releasable pool of synaptic vesicles. *J. Neurosci.* 32 68–84. 10.1523/JNEUROSCI.3215-11.2012 22219271 PMC6621323

[B63] Gospodarowicz D. Greenburg G. Birdwell C. R. (1978). Determination of Cellular Shape by the Extracellular Matrix and Its Correlation with the Control of Cellular Growth. *Cancer Res.* 38 4155–4171. 359133

[B64] Grant S. G. N. Karl K. A. Kiebler M. A. Kandel E. R. (1995). Focal adhesion kinase in the brain: novel subcellular localization and specific regulation by Fyn tyrosine kinase in mutant mice. *Genes Dev.* 9 1909–1921. 10.1101/gad.9.15.1909 7544314

[B65] Grundy T. J. De Leon E. Griffin K. R. Stringer B. W. Day B. W. Fabry B. (2016). Differential response of patient-derived primary glioblastoma cells to environmental stiffness. *Sci. Rep.* 6:23353. 10.1038/srep23353 26996336 PMC4800394

[B66] Guan R. Xu X. Chen M. Hu H. Ge H. Wen S. (2013). Advances in the studies of roles of Rho/Rho-kinase in diseases and the development of its inhibitors. *Eur. J. Med. Chem.* 70 613–622. 10.1016/j.ejmech.2013.10.048 24211637

[B67] Hain E. G. Klein C. Munder T. Braun J. Riek K. Mueller S. (2016). Dopaminergic Neurodegeneration in the Mouse Is Associated with Decrease of Viscoelasticity of Substantia Nigra Tissue. *PLoS One* 11:e0161179. 10.1371/journal.pone.0161179 27526042 PMC4985068

[B68] Hall C. M. Moeendarbary E. Sheridan G. K. (2021). Mechanobiology of the brain in ageing and Alzheimer’s disease. *Eur. J. Neurosci.* 53 3851–3878. 10.1111/ejn.14766 32356339

[B69] Han M. K. de Rooij J. (2016). Converging and Unique Mechanisms of Mechanotransduction at Adhesion Sites. *Trends Cell Biol.* 26 612–623. 10.1016/j.tcb.2016.03.005 27036655

[B70] Hattori T. Yuasa T. Aoki S. Sato R. Sawaura H. Mori T. (2011). Altered microstructure in corticospinal tract in idiopathic normal pressure hydrocephalus: comparison with Alzheimer disease and Parkinson disease with dementia. *Am. J. Neuroradiol.* 32 1681–1687. 10.3174/ajnr.A2570 21816921 PMC7965405

[B71] Hegedus B. Marga F. Jakab K. Sharpe-Timms K. L. Forgacs G. (2006). The interplay of cell-cell and cell-matrix interactions in the invasive properties of brain tumors. *Biophys. J.* 91 2708–2716. 10.1529/biophysj.105.077834 16829558 PMC1562379

[B72] Heijl A. Leske M. C. Bengtsson B. Hyman L. Bengtsson B. Hussein M. (2002). Reduction of intraocular pressure and glaucoma progression: results from the Early Manifest Glaucoma trial. *Arch. Ophthalmol.* 120 1268–1279. 10.1001/archopht.120.10.1268 12365904

[B73] Hensel N. Rademacher S. Claus P. (2015). Chatting with the neighbors: crosstalk between Rho-kinase (ROCK) and other signaling pathways for treatment of neurological disorders. *Front. Neurosci.* 9:198. 10.3389/fnins.2015.00198 26082680 PMC4451340

[B74] Hoffman B. D. Grashoff C. Schwartz M. A. (2011). Dynamic molecular processes mediate cellular mechanotransduction. *Nature* 475 316–323. 10.1038/nature10316 21776077 PMC6449687

[B75] Hoshina N. Tezuka T. Yokoyama K. Kozuka-hata H. Oyama M. Yamamoto T. (2007). Focal adhesion kinase regulates laminin-induced oligodendroglial process outgrowth. *Genes Cells* 12 1245–1254. 10.1111/j.1365-2443.2007.01130.x 17986008

[B76] Hsieh H.-L. Wang H.-H. Wu W.-B. Chu P.-J. Yang C.-M. (2010). Transforming growth factor-β1 induces matrix metalloproteinase-9 and cell migration in astrocytes: roles of ROS-dependent ERK- and JNK-NF-κB pathways. *J. Neuroinflammation* 7:88. 10.1186/1742-2094-7-88 21134288 PMC3002339

[B77] Hu H. Z. Granger N. Pai S. B. Bellamkonda R. V. Jeffery N. D. (2018). Therapeutic efficacy of microtube-embedded chondroitinase ABC in a canine clinical model of spinal cord injury. *Brain* 141 1017–1027. 10.1093/brain/awy007 29444239

[B78] Huang A. S. Minasyan L. Weinreb R. N. (2017). Glaucoma-Intraocular Pressure Reduction. *Handb. Exp. Pharmacol.* 242 181–207. 10.1007/164_2016_24 27812895

[B79] Humphries J. D. Byron A. Humphries M. J. (2006). Integrin ligands at a glance. *J. Cell Sci.* 119 3901–3903. 10.1242/jcs.03098 16988024 PMC3380273

[B80] Imamura K. Takeshima T. Fusayasu E. Nakashima K. (2008). Increased plasma matrix metalloproteinase-9 levels in migraineurs. *Headache* 48 135–139. 10.1111/j.1526-4610.2007.00958.x 18005141

[B81] Jagielska A. Lowe A. L. Makhija E. Wroblewska L. Guck J. Franklin R. J. M. (2017). Mechanical Strain Promotes Oligodendrocyte Differentiation by Global Changes of Gene Expression. *Front. Cell. Neurosci.* 11:93. 10.3389/fncel.2017.00093 28473753 PMC5397481

[B82] Jagielska A. Norman A. L. Whyte G. Vliet K. J. V. Guck J. Franklin R. J. M. (2012). Mechanical environment modulates biological properties of oligodendrocyte progenitor cells. *Stem Cells Dev.* 21 2905–2914. 10.1089/scd.2012.0189 22646081 PMC5915215

[B83] Jiang X. Georges P. C. Li B. Du Y. Kutzing M. K. Previtera M. L. (2007). Cell Growth in Response to Mechanical Stiffness is Affected by Neuron-Astroglia Interactions. *Open Neurosci. J.* 1 7–14. 10.2174/1874082000701010007

[B84] Jin J. Zhao X. Fu H. Gao Y. (2020). The Effects of YAP and Its Related Mechanisms in Central Nervous System Diseases. *Front. Neurosci.* 14:595–595. 10.3389/fnins.2020.00595 32676008 PMC7333666

[B85] Kass M. A. Heuer D. K. Higginbotham E. J. Johnson C. A. Keltner J. L. Miller J. P. (2002). The ocular hypertension treatment study: a randomized trial determines that topical ocular hypotensive medication delays or prevents the onset of primary open-angle glaucoma. *Arch. Ophthalmol.* 120 701–713; discussion 829–730. 10.1001/archopht.120.6.701 12049574

[B86] Keating C. E. Cullen D. K. (2021). Mechanosensation in traumatic brain injury. *Neurobiol. Dis.* 148:105210. 10.1016/j.nbd.2020.105210 33259894 PMC7847277

[B87] Kemmerling N. Wunderlich P. Theil S. Linnartz-Gerlach B. Hersch N. Hoffmann B. (2017). Intramembranous processing by gamma-secretase regulates reverse signaling of ephrin-B2 in migration of microglia. *Glia* 65 1103–1118. 10.1002/glia.23147 28370426

[B88] Keung A. J. de Juan-Pardo E. M. Schaffer D. V. Kumar S. (2011). Rho GTPases mediate the mechanosensitive lineage commitment of neural stem cells. *Stem Cells* 29 1886–1897. 10.1002/stem.746 21956892 PMC5990277

[B89] Kilpatrick T. J. Ortuno D. Bucci T. Lai C. Lemke G. (2000). Rat oligodendroglia express c-met and focal adhesion kinase, protein tyrosine kinases implicated in regulating epithelial cell motility. *Neurosci. Lett.* 279 5–8. 10.1016/s0304-3940(99)00928-3 10670774

[B90] Kimura K. Ito M. Amano M. Chihara K. Fukata Y. Nakafuku M. (1996). Regulation of myosin phosphatase by Rho and Rho-associated kinase (Rho-kinase). *Science* 273 245–248. 8662509 10.1126/science.273.5272.245

[B91] Kippert A. Fitzner D. Helenius J. Simons M. (2009). Actomyosin contractility controls cell surface area of oligodendrocytes. *BMC Cell Biol.* 10:71. 10.1186/1471-2121-10-71 19781079 PMC2760528

[B92] Klein T. Bischoff R. (2011). Physiology and pathophysiology of matrix metalloproteases. *Amino Acids* 41 271–290. 10.1007/s00726-010-0689-x 20640864 PMC3102199

[B93] Krizbai I. A. Deli M. A. (2003). Signalling pathways regulating the tight junction permeability in the blood-brain barrier. *Cell Mol. Biol.* 49 23–31. 12839334

[B94] Kulkarni R. N. Bakker A. D. Gruber E. V. Chae T. D. Veldkamp J. B. Klein-Nulend J. (2012). MT1-MMP modulates the mechanosensitivity of osteocytes. *Biochem. Biophys. Res. Commun.* 417 824–829. 10.1016/j.bbrc.2011.12.045 22202174

[B95] Lahoria R. Allport L. Glenn D. Masters L. Shnier R. Davies M. (2012). Spontaneous low pressure headache - A review and illustrative patient. *J. Clin. Neurosci.* 19 1076–1079. 10.1016/j.jocn.2011.12.014 22705138

[B96] Lakhan S. E. Avramut M. (2012). Matrix metalloproteinases in neuropathic pain and migraine: friends, enemies, and therapeutic targets. *Pain Res. Treat.* 2012:952906. 10.1155/2012/952906 22970361 PMC3434407

[B97] Lantoine J. Grevesse T. Villers A. Delhaye G. Mestdagh C. Versaevel M. (2016). Matrix stiffness modulates formation and activity of neuronal networks of controlled architectures. *Biomaterials* 89 14–24. 10.1016/j.biomaterials.2016.02.041 26946402

[B98] Lehoux S. Tedgui A. (2003). Cellular mechanics and gene expression in blood vessels. *J. Biomech.* 36 631–643. 10.1016/s0021-9290(02)00441-4 12694993

[B99] Leipzig N. D. Shoichet M. S. (2009). The effect of substrate stiffness on adult neural stem cell behavior. *Biomaterials* 30 6867–6878. 10.1016/j.biomaterials.2009.09.002 19775749

[B100] Lu Y. B. Franze K. Seifert G. Steinhauser C. Kirchhoff F. Wolburg H. (2006). Viscoelastic properties of individual glial cells and neurons in the CNS. *Proc. Natl. Acad. Sci. USA.* 103 17759–17764. 10.1073/pnas.0606150103 17093050 PMC1693820

[B101] Luo J. Li P. (2022). Context-dependent transcriptional regulations of YAP/TAZ in stem cell and differentiation. *Stem Cell Res. Therapy* 13:10. 10.1186/s13287-021-02686-y 35012640 PMC8751096

[B102] Ma Y. Gong Y. Cheng Z. Loganathan S. Kao C. Sarkaria J. N. (2015). Critical functions of RhoB in support of glioblastoma tumorigenesis. *Neuro Oncol.* 17 516–525. 10.1093/neuonc/nou228 25216671 PMC4483068

[B103] Maldonado H. Calderon C. Burgos-Bravo F. Kobler O. Zuschratter W. Ramirez O. (2017). Astrocyte-to-neuron communication through integrin-engaged Thy-1/CBP/Csk/Src complex triggers neurite retraction via the RhoA/ROCK pathway. *Biochim. Biophys. Acta Mol. Cell Res.* 1864 243–254. 10.1016/j.bbamcr.2016.11.006 27842221

[B104] Marta G. N. Correa S. F. Teixeira M. J. (2011). Meningioma: review of the literature with emphasis on the approach to radiotherapy. *Expert Rev. Anticancer Ther.* 11 1749–1758. 10.1586/era.11.162 22050024

[B105] Martin K. H. Slack J. K. Boerner S. A. Martin C. C. Parsons J. T. (2002). Integrin connections map: to infinity and beyond. *Science* 296 1652–1653. 10.1126/science.296.5573.1652 12040184

[B106] Martins-Oliveira A. Speciali J. G. Dach F. Marcaccini A. M. Goncalves F. M. Gerlach R. F. (2009). Different circulating metalloproteinases profiles in women with migraine with and without aura. *Clin. Chim. Acta* 408 60–64. 10.1016/j.cca.2009.07.008 19627981

[B107] Mathewson A. J. Berry M. (1985). Observations on the astrocyte response to a cerebral stab wound in adult rats. *Brain Res.* 327 61–69. 10.1016/0006-8993(85)91499-4 3986520

[B108] Matthews B. D. Overby D. R. Mannix R. Ingber D. E. (2006). Cellular adaptation to mechanical stress: role of integrins, Rho, cytoskeletal tension and mechanosensitive ion channels. *J. Cell Sci.* 119(Pt 3), 508–518. 10.1242/jcs.02760 16443749

[B109] May A. Bahra A. Buchel C. Frackowiak R. S. J. Goadsby P. J. (2000). PET and MRA findings in cluster headache and MRA in experimental pain. *Neurology* 55 1328–1335. 10.1212/wnl.55.9.1328 11087776

[B110] McBrien N. A. Jobling A. I. Gentle A. (2009). Biomechanics of the sclera in myopia: extracellular and cellular factors. *Optometry Vis. Sci.* 86 E23–E30. 10.1097/OPX.0b013e3181940669 19104466

[B111] McCawley L. J. Matrisian L. M. (2001). Matrix metalloproteinases: they’re not just for matrix anymore! *Curr. Opin. Cell Biol.* 13 534–540. 10.1016/s0955-0674(00)00248-9 11544020

[B112] McGeer P. L. Guo J. P. Lee M. Kennedy K. McGeer E. G. (2018). Alzheimer’s Disease Can Be Spared by Nonsteroidal Anti-Inflammatory Drugs. *J. Alzheimer’s Dis.* 62 1219–1222. 10.3233/JAD-170706 29103042 PMC5870017

[B113] Mikhailov N. Mamontov O. V. Kamshilin A. A. Giniatullin R. (2017). Parasympathetic Cholinergic and Neuropeptide Mechanisms of Migraine. *Anesth Pain Med.* 7:e42210. 10.5812/aapm.42210 28920040 PMC5554415

[B114] Min S. K. Kim S. H. Kim C. R. Paik S.-M. Jung S.-M. Shin H. S. (2013). Effect of topography of an electrospun nanofiber on modulation of activity of primary rat astrocytes. *Neurosci. Lett.* 534 80–84. 10.1016/j.neulet.2012.11.015 23178191

[B115] Mitra S. K. Hanson D. A. Schlaepfer D. D. (2005). Focal adhesion kinase: in command and control of cell motility. *Nat. Rev. Mol. Cell Biol.* 6 56–68. 10.1038/nrm1549 15688067

[B116] Miyamoto Y. Yamauchi J. Chan J. R. Okada A. Tomooka Y. Hisanaga S. I. (2007). Cdk5 regulates differentiation of oligodendrocyte precursor cells through the direct phosphorylation of paxillin. *J. Cell Sci.* 120 4355–4366. 10.1242/jcs.018218 18042622

[B117] Moore S. W. Zhang X. Lynch C. D. Sheetz M. P. (2012). Netrin-1 Attracts axons through FAK-dependent mechanotransduction. *J. Neurosci.* 32 11574–11585. 10.1523/JNEUROSCI.0999-12.2012 22915102 PMC3461192

[B118] MORANTE J. Desplan C. (2004). Building a projection map for photoreceptor neurons in the *Drosophila* optic lobes. *Semin. Cell Dev. Biol.* 15 137–143. 10.1016/j.semcdb.2003.09.007 15036216

[B119] Morgan J. T. Murphy C. J. Russell P. (2013). What do mechanotransduction, Hippo, Wnt, and TGFβ have in common? YAP and TAZ as key orchestrating molecules in ocular health and disease. *Exp. Eye Res.* 2013:12. 10.1016/j.exer.2013.06.012 23792172 PMC3795947

[B120] Morgenstern D. A. Asher R. A. Fawcett J. W. (2002). Chondroitin sulphate proteoglycans in the CNS injury response. *Prog. Brain Res.* 137 313–332. 10.1016/s0079-6123(02)37024-9 12440375

[B121] Moses G. S. D. Jensen M. D. Lue L. F. Walker D. G. Sun A. Y. Simonyi A. (2006). Secretory PLA2-IIA: a new inflammatory factor for Alzheimer’s disease. *J. Neuroinflamm.* 3:28. 10.1186/1742-2094-3-28 17026770 PMC1613236

[B122] Moshayedi P. Da F. Costa L. Christ A. Lacour S. P. Fawcett J. (2010). Mechanosensitivity of astrocytes on optimized polyacrylamide gels analyzed by quantitative morphometry. *J. Phys. Condensed Matter* 22:194114. 10.1088/0953-8984/22/19/194114 21386440

[B123] Mroczko B. Groblewska M. Barcikowska M. (2013). The role of matrix metalloproteinases and tissue inhibitors of metalloproteinases in the pathophysiology of neurodegeneration: a literature study. *J. Alzheimers Dis.* 37 273–283. 10.3233/jad-130647 23792694

[B124] Munder T. Pfeffer A. Schreyer S. Guo J. Braun J. Sack I. (2018). MR elastography detection of early viscoelastic response of the murine hippocampus to amyloid beta accumulation and neuronal cell loss due to Alzheimer’s disease. *J. Magn. Reson. Imaging* 47 105–114. 10.1002/jmri.25741 28422391

[B125] Murphy M. C. Curran G. L. Glaser K. J. Rossman P. J. Huston J. Poduslo J. F. (2012). Magnetic resonance elastography of the brain in a mouse model of Alzheimer’s disease: initial results. *Magn. Resonan. Imaging* 30 535–539. 10.1016/j.mri.2011.12.019 22326238 PMC3433281

[B126] Murphy M. C. Jones D. T. Jack C. R. Glaser K. J. Senjem M. L. Manduca A. (2016). Regional brain stiffness changes across the Alzheimer’s disease spectrum. *NeuroImage Clin.* 10 283–290. 10.1016/j.nicl.2015.12.007 26900568 PMC4724025

[B127] Nassini R. de Cesaris F. Pedretti P. Geppetti P. (2010). TRPS and migraine. *Open Drug Discov. J.* 2 55–63. 10.2174/1877381801002030055

[B128] Navarro A. I. Rico B. (2014). Focal adhesion kinase function in neuronal development. *Curr. Opin. Neurobiol.* 27 (Suppl. C), 89–95. 10.1016/j.conb.2014.03.002 24705242

[B129] Orgaz J. L. Herraiz C. Sanz-Moreno V. (2014). Rho GTPases modulate malignant transformation of tumor cells. *Small GTPases* 5:e983867. 10.4161/sgtp.29019 25036871 PMC4125382

[B130] Ostrow L. W. Sachs F. (2005). Mechanosensation and endothelin in astrocytes - Hypothetical roles in CNS pathophysiology. *Brain Res. Rev.* 48 488–508. 10.1016/j.brainresrev.2004.09.005 15914254

[B131] Page-McCaw A. Ewald A. J. Werb Z. (2007). Matrix metalloproteinases and the regulation of tissue remodelling. *Nat. Rev. Mol. Cell Biol.* 8 221–233. 10.1038/nrm2125 17318226 PMC2760082

[B132] Paparcone R. Pires M. A. Buehler M. J. (2010). Mutations Alter the Geometry and Mechanical Properties of Alzheimer’s Aβ(1-40) Amyloid Fibrils. *Biochemistry* 49 8967–8977. 10.1021/bi100953t 20731379

[B133] Pasqualetti P. Bonomini C. Dal Forno G. Sinforiani E. Marra C. (2009). A randomized controlled study on effects of ibuprofen on cognitive progression of Alzheimer’s disease. *Aging Clin. Exp. Res.* 21 102–110. 10.1007/BF03325217 19448381

[B134] Paterson K. Lolignier S. Wood J. N. McMahon S. B. Bennett D. L. H. (2014). Botulinum toxin-a treatment reduces human mechanical pain sensitivity and mechanotransduction. *Ann. Neurol.* 75 591–596. 10.1002/ana.24122 24550077 PMC4112716

[B135] Pepin K. M. Ehman R. L. McGee K. P. (2015). Magnetic resonance elastography (MRE) in cancer: technique, analysis, and applications. *Prog. Nuclear Magn. Resonan. Spectrosc.* 0 32–48. 10.1016/j.pnmrs.2015.06.001 26592944 PMC4660259

[B136] Pineda A. A. M. Minohara M. Kawamura N. Matsushita T. Yamasaki R. Sun X. (2011). Preventive and therapeutic effects of the selective Rho-kinase inhibitor fasudil on experimental autoimmune neuritis. *J. Neurol. Sci.* 306 115–120. 10.1016/j.jns.2011.03.031 21501850

[B137] Pires L. R. Pego A. P. (2015). Bridging the lesion - engineering a permissive substrate for nerve regeneration. *Regen. Biomater.* 2 203–214. 10.1093/rb/rbv012 26816642 PMC4669012

[B138] Pires L. R. Lopes C. D. F. Salvador D. Rocha D. N. Pego A. P. (2017). Ibuprofen-loaded fibrous patches-taming inhibition at the spinal cord injury site. *J. Mater. Sci. Mater. Med.* 28:157. 10.1007/s10856-017-5967-7 28894995

[B139] Pires L. R. Rocha D. N. Ambrosio L. Pêgo A. P. (2015). The role of the surface on microglia function: implications for central nervous system tissue engineering. *J. R. Soc. Interface* 6:1224. 10.1098/rsif.2014.1224 25540243 PMC4305425

[B140] Provenzano P. P. Inman D. R. Eliceiri K. W. Keely P. J. (2009). Matrix density-induced mechanoregulation of breast cell phenotype, signaling and gene expression through a FAK-ERK linkage. *Oncogene* 28 4326–4343. 10.1038/onc.2009.299 19826415 PMC2795025

[B141] Putnam A. J. Cunningham J. J. Pillemer B. B. L. Mooney D. J. (2003). External mechanical strain regulates membrane targeting of Rho GTPases by controlling microtubule assembly. *Am. J. Physiol. Cell Physiol.* 284 C627–C639. 10.1152/ajpcell.00137.2002 12409284

[B142] Qazi H. Shi Z. D. Tarbel J. M. (2011). Fluid shear stress regulates the invasive potential of glioma cells via modulation of migratory activity and matrix metalloproteinase expression. *PLoS One* 6:e20348. 10.1371/journal.pone.0020348 21637818 PMC3102715

[B143] Raad M. El Tal T. Gul R. Mondello S. Zhang Z. Boustany R. M. (2012). Neuroproteomics approach and neurosystems biology analysis: ROCK inhibitors as promising therapeutic targets in neurodegeneration and neurotrauma. *Electrophoresis* 33 3659–3668. 10.1002/elps.201200470 23161464

[B144] Raghow R. (1994). The role of extracellular matrix in postinflammatory wound healing and fibrosis. *FASEB J.* 8 823–831. 10.1096/fasebj.8.11.8070631 8070631

[B145] Rahkonen L. Rutanen E. M. Unkila-Kallio L. Nuutila M. Nieminen P. Sorsa T. (2009). Factors affecting matrix metalloproteinase-8 levels in the vaginal and cervical fluids in the first and second trimester of pregnancy. *Hum. Reprod.* 24 2693–2702. 10.1093/humrep/dep284 19654111

[B146] Rajasekharan S. Baker K. A. Horn K. E. Jarjour A. A. Antel J. P. Kennedy T. E. (2009). Netrin 1 and Dcc regulate oligodendrocyte process branching and membrane extension via Fyn and RhoA. *Development* 136 415–426. 10.1242/dev.018234 19141671

[B147] Ramón C. S. (1928). *Degeneration and Regeneration of the Nervous System.* Oxford: Oxford University Press.

[B148] Ren X. D. Kiosses W. B. Schwartz M. A. (1999). Regulation of the small GTP-binding protein Rho by cell adhesion and the cytoskeleton. *EMBO J.* 18 578–585. 10.1093/emboj/18.3.578 9927417 PMC1171150

[B149] Ribeiro A. R. Barbaglio A. Oliveira M. J. Ribeiro C. C. Wilkie I. C. Candia Carnevali M. D. (2012). Matrix metalloproteinases in a sea urchin ligament with adaptable mechanical properties. *PLoS One* 7:e49016. 10.1371/journal.pone.0049016 23173042 PMC3500250

[B150] Riek K. Millward J. M. Hamann I. Mueller S. Pfueller C. F. Paul F. (2012). Magnetic resonance elastography reveals altered brain viscoelasticity in experimental autoimmune encephalomyelitis. *NeuroImage Clin.* 1 81–90. 10.1016/j.nicl.2012.09.003 24179740 PMC3757734

[B151] Riveline D. Zamir E. Balaban N. Q. Schwarz U. S. Ishizaki T. Narumiya S. (2001). Focal contacts as mechanosensors: externally applied local mechanical force induces growth of focal contacts by an mDia1-dependent and ROCK-independent mechanism. *J. Cell Biol.* 153 1175–1185. 10.1083/jcb.153.6.1175 11402062 PMC2192034

[B152] Rocha D. N. Brites P. Fonseca C. Pego A. P. (2014). Poly(trimethylene carbonate-co-epsilon-caprolactone) promotes axonal growth. *PLoS One* 9:e88593. 10.1371/journal.pone.0088593 24586346 PMC3937290

[B153] Rocha D. N. Ferraz-Nogueira J. P. Barrias C. C. Relvas J. B. Pego A. P. (2015). Extracellular environment contribution to astrogliosis-lessons learned from a tissue engineered 3D model of the glial scar. *Front. Cell Neurosci.* 9:377. 10.3389/fncel.2015.00377 26483632 PMC4586948

[B154] Rosario Hernandez M. Pena J. D. O. (1997). The optic nerve head in glaucomatous optic neuropathy. *Arch. Ophthalmol.* 115 389–395. 10.1001/archopht.1997.01100150391013 9076213

[B155] Rosenberg G. A. (2009). Matrix metalloproteinases and their multiple roles in neurodegenerative diseases. *Lancet Neurol.* 8 205–216. 10.1016/S1474-4422(09)70016-X 19161911

[B156] Rosenzweig E. S. Salegio E. A. Liang J. J. Weber J. L. Weinholtz C. A. Brock J. H. (2019). Chondroitinase improves anatomical and functional outcomes after primate spinal cord injury. *Nat. Neurosci.* 22:1269. 10.1038/s41593-019-0424-1 31235933 PMC6693679

[B157] Roser A. E. Tonges L. Lingor P. (2017). Modulation of Microglial Activity by Rho-Kinase (ROCK) Inhibition as Therapeutic Strategy in Parkinson’s Disease and Amyotrophic Lateral Sclerosis. *Front. Aging Neurosci.* 9:94. 10.3389/fnagi.2017.00094 28420986 PMC5378706

[B158] Ross B. M. Moszczynska A. Erlich J. Kish S. J. (1998). Phospholipid-metabolizing enzymes in Alzheimer’s disease: increased lysophospholipid acyltransferase activity and decreased phospholipase A2 activity. *J. Neurochem.* 70 786–793. 10.1046/j.1471-4159.1998.70020786.x 9453575

[B159] Saha K. Keung A. J. Irwin E. F. Li Y. Little L. Schaffer D. V. (2008). Substrate modulus directs neural stem cell behavior. *Biophys. J.* 95 4426–4438. 10.1529/biophysj.108.132217 18658232 PMC2567955

[B160] Saxena T. Gilbert J. Stelzner D. Hasenwinkel J. (2012). Mechanical characterization of the injured spinal cord after lateral spinal hemisection injury in the rat. *J. Neurotrauma* 29 1747–1757. 10.1089/neu.2011.1818 22435754

[B161] Schiller H. B. Hermann M. R. Polleux J. Vignaud T. Zanivan S. Friedel C. C. (2013). B1- and v-class integrins cooperate to regulate myosin II during rigidity sensing of bronectin-based microenvironments. *Nat. Cell Biol.* 15 625–636. 10.1038/ncb2747 23708002

[B162] Schregel K. Née Tysiak E. W. Garteiser P. Gemeinhardt I. Prozorovski T. Aktas O. (2012). Demyelination reduces brain parenchymal stiffness quantified in vivo by magnetic resonance elastography. *Proc. Natl. Acad. Sci. USA.* 109 6650–6655. 10.1073/pnas.1200151109 22492966 PMC3340071

[B163] Sen S. Dong M. Kumar S. (2009). Isoform-specific contributions of alpha-actinin to glioma cell mechanobiology. *PLoS One* 4:e8427. 10.1371/journal.pone.0008427 20037648 PMC2793025

[B164] Sen S. Ng W. P. Kumar S. (2012). Contributions of talin-1 to glioma cell-matrix tensional homeostasis. *J. R. Soc. Interface* 9 1311–1317. 10.1098/rsif.2011.0567 22158841 PMC3350720

[B165] Shreiber D. I. Hao H. Elias R. A. (2009). Probing the influence of myelin and glia on the tensile properties of the spinal cord. *Biomech. Model. Mechanobiol.* 8 311–321. 10.1007/s10237-008-0137-y 18719957

[B166] Sigal I. A. (2009). Interactions between geometry and mechanical properties on the optic nerve head. *Investig. Ophthalmol. Vis. Sci.* 50 2785–2795. 10.1167/iovs.08-3095 19168906

[B167] Sigal I. A. Ethier C. R. (2009). Biomechanics of the optic nerve head. *Exp. Eye Res.* 88 799–807. 19217902 10.1016/j.exer.2009.02.003

[B168] Sivak J. M. Fini M. E. (2002). MMPs in the eye: emerging roles for matrix metalloproteinases in ocular physiology. *Prog. Retin. Eye Res.* 21 1–14. 10.1016/s1350-9462(01)00015-5 11906808

[B169] Sivasankaran R. Pei J. Wang K. C. Zhang Y. P. Shields C. B. Xu X. M. (2004). PKC mediates inhibitory effects of myelin and chondroitin sulfate proteoglycans on axonal regeneration. *Nat. Neurosci.* 7 261–268. 10.1038/nn1193 14770187

[B170] Sloane J. A. Batt C. Ma Y. Harris Z. M. Trapp B. Vartanian T. (2010). Hyaluronan blocks oligodendrocyte progenitor maturation and remyelination through TLR2. *Proc. Natl. Acad. Sci. USA.* 107 11555–11560. 10.1073/pnas.1006496107 20534434 PMC2895128

[B171] Steinmetz M. P. Horn K. P. Tom V. J. Miller J. H. Busch S. A. Nair D. (2005). Chronic enhancement of the intrinsic growth capacity of sensory neurons combined with the degradation of inhibitory proteoglycans allows functional regeneration of sensory axons through the dorsal root entry zone in the mammalian spinal cord. *J. Neurosci.* 25 8066–8076. 10.1523/JNEUROSCI.2111-05.2005 16135764 PMC6725461

[B172] Sternlicht M. D. Werb Z. (2001). How matrix metalloproteinases regulate cell behavior. *Annu. Rev. Cell Dev. Biol.* 17 463–516. 10.1146/annurev.cellbio.17.1.463 11687497 PMC2792593

[B173] Streitberger K. Sack I. Krefting D. Pfüller C. Braun J. Paul F. (2012). Brain viscoelasticity alteration in chronic-progressive multiple sclerosis. *PLoS One* 7:e29888–e29888. 10.1371/journal.pone.0029888 22276134 PMC3262797

[B174] Strouthidis N. G. Girard M. J. A. (2013). Altering the way the optic nerve head responds to intraocular pressure - A potential approach to glaucoma therapy. *Curr. Opin. Pharmacol.* 13 83–89. 10.1016/j.coph.2012.09.001 22999652

[B175] Sun Y. Xu S. Jiang M. Liu X. Yang L. Bai Z. (2021). Role of the Extracellular Matrix in Alzheimer’s Disease. *Front. Aging Neurosci.* 13:707466. 10.3389/fnagi.2021.707466 34512308 PMC8430252

[B176] Swaminathan V. Mythreye K. O’Brien E. T. Berchuck A. Blobe G. C. Superfine R. (2011). Mechanical stiffness grades metastatic potential in patient tumor cells and in cancer cell lines. *Cancer Res.* 71 5075–5080. 10.1158/0008-5472.CAN-11-0247 21642375 PMC3220953

[B177] Sweers K. K. M. Bennink M. L. Subramaniam V. (2012). Nanomechanical properties of single amyloid fibrils. *J. Phys. Condensed Matter* 24:243101. 10.1088/0953-8984/24/24/243101 22585542

[B178] Tan H. B. Zhong Y. S. Cheng Y. Shen X. (2011). Rho/ROCK pathway and neural regeneration: a potential therapeutic target for central nervous system and optic nerve damage. *Int. J. Ophthalmol.* 4 652–657. 10.3980/j.issn.2222-3959.2011.06.16 22553739 PMC3340788

[B179] Tanaka H. Homma H. Fujita K. Kondo K. Yamada S. Jin X. (2020). YAP-dependent necrosis occurs in early stages of Alzheimer’s disease and regulates mouse model pathology. *Nat. Commun.* 11:507. 10.1038/s41467-020-14353-6 31980612 PMC6981281

[B180] Tonges L. Frank T. Tatenhorst L. Saal K. A. Koch J. C. Szego E. M. (2012). Inhibition of rho kinase enhances survival of dopaminergic neurons and attenuates axonal loss in a mouse model of Parkinson’s disease. *Brain* 135 3355–3370. 10.1093/brain/aws254 23087045 PMC3501973

[B181] Ulrich T. A. de Juan Pardo E. M. Kumar S. (2009). The mechanical rigidity of the extracellular matrix regulates the structure, motility, and proliferation of glioma cells. *Cancer Res.* 69 4167–4174. 10.1158/0008-5472.CAN-08-4859 19435897 PMC2727355

[B182] Ungureanu A.-A. Benilova I. Krylychkina O. Braeken D. De Strooper B. Van Haesendonck C. (2016). Amyloid beta oligomers induce neuronal elasticity changes in age-dependent manner: a force spectroscopy study on living hippocampal neurons. *Sci. Rep.* 6 25841–25841. 10.1038/srep25841 27173984 PMC4865860

[B183] Urbanski M. M. Brendel M. B. Melendez-Vasquez C. V. (2019). Acute and chronic demyelinated CNS lesions exhibit opposite elastic properties. *Sci. Rep.* 9:999. 10.1038/s41598-018-37745-7 30700777 PMC6354022

[B184] Van den Eynde C. De Clercq K. Vriens J. (2021). Transient Receptor Potential Channels in the Epithelial-to-Mesenchymal Transition. *Int. J. Mol. Sci.* 22:22158188. 10.3390/ijms22158188 34360952 PMC8348042

[B185] Virchow R. (1858). Cellular pathology. As based upon physiological and pathological histology. Lecture XVI–Atheromatous affection of arteries. 1858. *Nutr. Rev.* 47 23–25. 10.1111/j.1753-4887.1989.tb02747.x 2649802

[B186] Vlodavsky I. Levi A. Lax I. Fuks Z. Schlessinger J. (1982). Induction of cell attachment and morphological differentiation in a pheochromocytoma cell line and embryonal sensory cells by the extracellular matrix. *Dev. Biol.* 93 285–300. 10.1016/0012-1606(82)90118-x 7141098

[B187] Vogel V. Sheetz M. (2006). Local force and geometry sensing regulate cell functions. *Nat. Rev. Mol. Cell Biol.* 7 265–275. 10.1038/nrm1890 16607289

[B188] Wang C. Tong X. Yang F. (2014). Bioengineered 3D brain tumor model to elucidate the effects of matrix stiffness on glioblastoma cell behavior using PEG-based hydrogels. *Mol. Pharm.* 11 2115–2125. 10.1021/mp5000828 24712441

[B189] Wang L. You X. Lotinun S. Zhang L. Wu N. Zou W. (2020). Mechanical sensing protein PIEZO1 regulates bone homeostasis via osteoblast-osteoclast crosstalk. *Nat. Commun.* 11:282. 10.1038/s41467-019-14146-6 31941964 PMC6962448

[B190] Wang N. Butler J. P. Ingber D. E. (1993). Mechanotransduction across the cell surface and through the cytoskeleton. *Science* 260 1124–1127. 10.1126/science.7684161 7684161

[B191] Webb A. Clark P. Skepper J. Compston A. Wood A. (1995). Guidance of oligodendrocytes and their progenitors by substratum topography. *J. Cell Sci.* 108(Pt 8), 2747–2760. 10.1242/jcs.108.8.2747 7593316

[B192] Weiss P. (1934). In vitro experiments on the factors determining the course of the outgrowing nerve fiber. *J. Exp. Zool.* 68 393–448. 10.1002/jez.1400680304

[B193] Wilson C. L. Hayward S. L. Kidambi S. (2016). Astrogliosis in a dish: substrate stiffness induces astrogliosis in primary rat astrocytes. *RSC Adv.* 6 34447–34457. 10.1039/C5RA25916A 32742641 PMC7394306

[B194] Wong S. Y. Ulrich T. A. Deleyrolle L. P. MacKay J. L. Lin J. M. Martuscello R. T. (2015). Constitutive activation of myosin-dependent contractility sensitizes glioma tumor-initiating cells to mechanical inputs and reduces tissue invasion. *Cancer Res.* 75 1113–1122. 10.1158/0008-5472.can-13-3426 25634210 PMC4359960

[B195] Woo S. Gomez T. M. (2006). Rac1 and RhoA promote neurite outgrowth through formation and stabilization of growth cone point contacts. *J. Neurosci.* 26 1418–1428. 10.1523/JNEUROSCI.4209-05.2006 16452665 PMC6675502

[B196] Wozniak M. A. Desai R. Solski P. A. Der C. J. Keely P. J. (2003). ROCK-generated contractility regulates breast epithelial cell differentiation in response to the physical properties of a three-dimensional collagen matrix. *J. Cell Biol.* 163 583–595. 10.1083/jcb.200305010 14610060 PMC2173660

[B197] Xu Z. Paparcone R. Buehler M. J. (2010). Alzheimer’s Ab(1-40) Amyloid Fibrils Feature Size-Dependent Mechanical Properties. *Biophys. J.* 98 2053–2062. 10.1016/j.bpj.2009.12.4317 20483312 PMC2872369

[B198] Yamada S. Ishikawa M. Yamaguchi M. Yamamoto K. (2019). Longitudinal morphological changes during recovery from brain deformation due to idiopathic normal pressure hydrocephalus after ventriculoperitoneal shunt surgery. *Sci. Rep.* 9:17318. 10.1038/s41598-019-53888-7 31754171 PMC6872815

[B199] Yamaguchi H. Wyckoff J. Condeelis J. (2005). Cell migration in tumors. *Curr. Opin. Cell Biol.* 17 559–564. 10.1016/j.ceb.2005.08.002 16098726

[B200] Yang X. Askarova S. Lee J. C. M. (2010). Membrane biophysics and mechanics in alzheimer’s disease. *Mol. Neurobiol.* 41 138–148. 10.1007/s12035-010-8121-9 20437210

[B201] Yong V. W. (2005). Metalloproteinases: mediators of pathology and regeneration in the CNS. *Nat. Rev. Neurosci.* 6 931–944. 10.1038/nrn1807 16288297

[B202] Zhu L. Su Q. Jie X. Liu A. Wang H. He B. (2016). NG2 expression in microglial cells affects the expression of neurotrophic and proinflammatory factors by regulating FAK phosphorylation. *Sci. Rep.* 6:27983. 10.1038/srep27983 27306838 PMC4910048

